# Multi-Study Proteomic and Bioinformatic Identification of Molecular Overlap between Amyotrophic Lateral Sclerosis (ALS) and Spinal Muscular Atrophy (SMA)

**DOI:** 10.3390/brainsci8120212

**Published:** 2018-12-04

**Authors:** Darija Šoltić, Melissa Bowerman, Joanne Stock, Hannah K. Shorrock, Thomas H. Gillingwater, Heidi R. Fuller

**Affiliations:** 1 School of Medicine, Keele University, Staffordshire ST5 5BG, UK; d.soltic@keele.ac.uk (D.S.); m.bowerman@keele.ac.uk (M.B.); 2 Institute for Science and Technology in Medicine, Keele University, Staffordshire ST5 5BG, UK; 3 Wolfson Centre for Inherited Neuromuscular Disease, RJAH Orthopaedic Hospital, Oswestry SY10 7AG, UK; 4 Biomedical Sciences, Edinburgh Medical School, University of Edinburgh, Edinburgh EH8 9AG, UK; hannah.shorrock@ufl.edu (H.K.S.); t.gillingwater@ed.ac.uk (T.H.G.); 5 Euan MacDonald Centre for Motor Neurone Disease Research, University of Edinburgh, Edinburgh EH8 9AG, UK

**Keywords:** spinal muscular atrophy (SMA), amyotrophic lateral sclerosis (ALS), proteomics, bioinformatics, endoplasmic reticulum-Golgi trafficking, ER-Golgi trafficking, calreticulin (CALR), endoplasmic reticulum, calcium

## Abstract

Unravelling the complex molecular pathways responsible for motor neuron degeneration in amyotrophic lateral sclerosis (ALS) and spinal muscular atrophy (SMA) remains a persistent challenge. Interest is growing in the potential molecular similarities between these two diseases, with the hope of better understanding disease pathology for the guidance of therapeutic development. The aim of this study was to conduct a comparative analysis of published proteomic studies of ALS and SMA, seeking commonly dysregulated molecules to be prioritized as future therapeutic targets. Fifteen proteins were found to be differentially expressed in two or more proteomic studies of both ALS and SMA, and bioinformatics analysis identified over-representation of proteins known to associate in vesicles and molecular pathways, including metabolism of proteins and vesicle-mediated transport—both of which converge on endoplasmic reticulum (ER)-Golgi trafficking processes. Calreticulin, a calcium-binding chaperone found in the ER, was associated with both pathways and we independently confirm that its expression was decreased in spinal cords from SMA and increased in spinal cords from ALS mice. Together, these findings offer significant insights into potential common targets that may help to guide the development of new therapies for both diseases.

## 1. Introduction

The term “motor neuron disease” incorporates several conditions, including the most common adult form, amyotrophic lateral sclerosis (ALS), and the predominantly childhood form, spinal muscular atrophy (SMA); both of which represent a devastating cause of disability, morbidity, and mortality. ALS is clinically characterized by muscular atrophy due to loss of motor neurons in the anterior horn of the spinal cord (“lower motor neurons”) and spasticity due to loss of cortical neurons (“upper motor neurons”) [1]. While approximately 90% of ALS cases occur sporadically (sALS), the remaining 10% of cases are attributable to a familial form of ALS (fALS) that typically follow an autosomal dominant inheritance pattern [2]. Genetic mutations have been identified accounting for half of the fALS cases, including mutations in the superoxide dismutase 1 (*SOD1*) gene, the TAR DNA Binding protein-43 (*TDP-43*) gene, the fused-in-sarcoma (*FUS*) gene, and a hexonucleotide repeat within the *C9orf72* gene [3]. SMA is also primarily characterized by loss of lower motor neurons and subsequent muscular atrophy, but without upper motor neuron involvement [4]. For >95% of SMA patients, the disease is caused by a loss-of-function defect in the *SMN1* gene, resulting in reduced levels of the ubiquitously-expressed survival of motor neuron (SMN) protein [5]. 

Unravelling the genetic basis of SMA and a proportion of ALS cases has been influential in guiding the direction of research, allowing for the development of animal models to further expand the study of motor neuron diseases. There is no cure for either disease, though some progress has been made in recent years towards the development of therapies that may extend survival or alleviate symptoms (reviewed for SMA [6], and for ALS [7]). Despite numerous clinical trials for ALS therapies, the only clinically approved treatments are riluzole, which may prolong survival by a modest 2–3 months, and edaravone, which was recently approved by the FDA to slow disease progression [8]. In 2017, nusinersen received clinical approval as the first antisense oligonucleotide gene therapy for SMA treatment. Clinical trial data suggests that treatment with nusinersen significantly improved disease symptoms for approximately 50% in patients with the most severe form of SMA (SMA Type I) [9,10], with a more modest effect in older patients [9,10,11]. There is a keen interest, therefore, to find alternative approaches to SMA and ALS therapy. For SMA, the need to tailor these treatments to the stage of development, the severity of disease, and to the effects of aging is widely recognized, but development has been hindered by lack of understanding of the mechanisms involved in disease pathogenesis and progression [6]. The disease mechanisms of ALS are likely to be even more complex, especially considering that approximately 90% of cases still have an unexplained etiology. 

Despite variation in the genetic basis of fALS and sALS, there is little difference recognized in their clinical presentation (aside from younger age at presentation in fALS) or any phenotypic pattern related to different genes [1,12]. This would suggest that the disease mechanisms of ALS likely converge on common molecular pathways, regardless of the etiology, that ultimately result in similar phenotypes. This then raises the question of whether there are core regulatory pathways involved in motor neuron disease pathology, in general, that may prove useful for guiding research into therapeutic development. Not unsurprisingly, therefore, the potential molecular similarities between ALS and SMA are receiving attention. Several recent reviews have discussed the current knowledge of pathways upon which both diseases appear to converge, including RNA processing pathways [13,14] and the TRAF6-nuclear factor kappa B (NF-κB) pathway [15]. Whilst these pathways certainly warrant further investigation, the precise molecular overlap between ALS and SMA is yet to be determined. 

Quantitative proteomics technology is well equipped to examine the complex molecular biology of both ALS and SMA and has been employed by research groups to study a range of cells and tissues from patients and animal models. Generation of these big datasets, however, has typically been followed by selection of several candidates for further examination, while most of the detected molecular changes have received little or no further attention. As a result, it is quite likely that important molecular changes have been overlooked, particularly at the level of molecular networks and pathways. Indeed, we previously interrogated published SMA proteomic datasets, and identified several proteins that were consistently dysregulated across multiple studies that had not previously been studied in association with SMA [16]. Importantly, this approach also highlighted conserved molecular responses to reduced SMN already proven to be directly relevant to SMA disease pathogenesis [16]. This provides confidence that multi-study comparison of proteomics data is a valid approach to finding disease-relevant targets for therapy design.

Here, we have applied the same approach to interrogate a range of ALS proteomic datasets to gain new insights into mechanisms of disease pathogenesis. By systematically comparing the resulting pooled dataset with the previously published dataset of conserved molecular alterations in SMA [16], we highlight the core molecular overlap between both diseases and use bioinformatics tools to understand the pathways upon which they converge. In addition, we report that calreticulin, a calcium-binding chaperone involved in the regulation of nascent protein folding [17], is associated with several perturbed pathways and we confirm that its expression levels are altered in spinal cords from both SMA and ALS mice.

## 2. Materials and Methods

### 2.1. Identification of ALS Proteomic Studies

PubMed searches were performed to identify eligible studies for comparison, using combinations of the terms: “amyotrophic lateral sclerosis”, “Lou Gehrig’s disease” or “motor neuron disease” AND “proteomics” or “mass spectrometry”. The search was conducted to include studies published up to 1 July 2018, and published studies were considered eligible for inclusion if they had applied an unbiased proteomics approach to identify disease-specific protein patterns in ALS. Studies that utilized a targeted approach (i.e., seeking specific proteins) for initial protein identification were excluded. Initially 40 studies were eligible for review, but seven studies were further excluded from the list due to inaccessible or incomplete data (i.e., lack of access to complete article, only some results published or accessible), leaving 33 studies in total. Of these, 12 studies examined proteome changes in biofluids from ALS patients and 21 studies examined proteome changes in cells and tissues from ALS models (Table S1).

### 2.2. Comparison of ALS Proteomic Studies

The studies eligible for review applied a variety of strategies in data analysis and processing, and so it was therefore necessary to establish a method of data selection for the comparison. First, only proteins changed in expression in ALS compared to healthy controls were considered for analysis. Proteins that were differentially expressed between ALS and diseased controls (e.g., Alzheimer’s disease (AD)), protein changes identified between different groups of ALS patients, and/or protein changes identified between different regions of the same tissue were disregarded. Results from studies that used multiple groups in proteomic comparison were filtered so that only proteins that showed differential expression between ALS and healthy controls were included in the comparison. Proteomic datasets were first extracted into a Microsoft Excel spreadsheet. To facilitate an achievable and accurate method for comparison between the studies the protein identifiers from each study were then converted to official gene symbols using the Database for Annotation, Visualization and Integrated Discovery (DAVID 6.8) [18,19]. If protein identifiers were not provided, or if DAVID could not recognize the accession number, identifiers were assigned manually by searching for protein names using the National Center for Biotechnology Information (NCBI) with the appropriate species filter to identify the corresponding accession number. Rarely, proteins had been removed from the NCBI database and so were not included in the final analysis. Most studies presented changes in the protein expression as a fold change (FC) difference between ALS and controls, after having applied a cut-off value to identify proteins that were differentially expressed in ALS. In cases where the cut-off value for fold change was not applied, only proteins that showed at least 20% change in the expression were included in the comparison. (This value was chosen because it has been commonly used in other published proteomic studies to identify differentially expressed proteins e.g., [20,21,22]). The final lists of gene names were then compared using Microsoft Excel’s ‘pivot table’ function to identify proteins that appeared in multiple studies.

### 2.3. Bioinformatics Analysis

The DAVID [18,19] platform was used to investigate the likely function of proteins that showed consistent change in expression across ALS studies. Gene ontology (GO) analysis was conducted to include terms that had at least three annotated proteins and a *p*-value ≤ 0.05. Redundant terms (i.e., those that were describing the same function and had identical matches, e.g., response to oxidative stress and removal of superoxide radicals) were combined into one term. Search Tool for the Retrieval of Interacting Genes/Proteins (STRING) 10 [23] was used to identify statistically significant interactions between proteins that showed consistent change in expression in ALS. Association network analysis was performed with high confidence (0.700) interaction score to exclude false positive results. 

### 2.4. Comparison of SMA and ALS Proteome Changes

Differentially expressed proteins identified in the present ALS comparison (Tables S2–S5) were compared with the differentially expressed proteins identified in our previous multi-study comparison of SMA proteomic datasets [16] to investigate common molecular patterns between the two diseases. Proteins with dysregulated expression in both SMA and ALS datasets were subjected to bioinformatics analysis using DAVID [18,19] and STRING 10 [23] as described above. The Reactome curated database [24] was then used to explore functional relationships between proteins by analyzing which pathways they map to and any known “reactions” within those pathways (i.e., individual steps in pathways that are known to change the state of a biological molecule). Reactome employs a binomial test to calculate the probability for each result, and *p*-values are corrected for the multiple testing using the Benjamini–Hochberg procedure.

### 2.5. Animal Models

For western blotting and immunohistochemistry experiments, the Taiwanese mouse model of severe SMA (*Smn^−/−^*; *SMN2^tg/o^*) on a congenic FVB background [25] and age-matched phenotypically normal controls (*Smn^+/−^*; *SMN2^tg/o^*) were obtained from breeding stock at the University of Edinburgh (Home Office license number PPL60/4569) and the ALS mouse model (*SOD1^G93A^*) [26] and 20-week-old age/gender-matched wild-type (WT) littermates were obtained from the University of Oxford (Home Office number PDFEDC6F0). The breeding strategy and genotyping using standard PCR protocols, were employed as previously described [27]. For quantitative PCR (qPCR) analysis, spinal cords were harvested from Taiwanese SMA (*Smn^−/−^*; *SMN2^tg/o^*) and control mice (*Smn^+/−^*; *SMN2^tg/o^*) as well as from 20-week-old *SOD1^G93A^* males and age/gender matched WT littermates housed at the University of Oxford (Home Office number PDFEDC6F0). All animal work was carried under the appropriate Project and Personal Licenses from the UK Home Office and following local ethical review.

### 2.6. Western Blotting

Spinal cords from postnatal day 8 (P8) SMA mice (*n* = 5) and healthy littermate mice (*n* = 5), and 20 week-old ALS mice (*n* = 5) and WT littermate mice (*n* = 4) were left on ice for 15 min in 2× modified RIPA buffer (2% NP-40, 0.5% Deoxycholic acid, 2 mM EDTA, 300 mM NaCl and 100 mM Tris-HCl (pH 7.4)), homogenised using pellet pestles and sonicated briefly 2–3 times every 10 min. Samples were centrifugated at 13,000 RPM (MSE, Heathfield, UK; MSB010.CX2.5 Micro Centaur) for 5 min at 4 °C to pellet any insoluble material. Protein extracts were subjected to SDS-PAGE and western blotting. Briefly, samples were boiled in 2× SDS sample buffer (4% SDS, 10% 2-mercaptoethanol, 20% glycerol, 0.125 M Tris-HCl (pH 6.8) and bromophenol blue) for 3 min at 95 °C and subjected to SDS-PAGE (Biorad, Hercules, CA, USA) using 4–12% Bis-Tris polyacrylamide gels (Life Technologies, Warrington, UK). A slice of the gel was excised and stained with Coomassie blue as an internal loading control for total protein. The proteins in the remaining part of the gel were transferred to nitrocellulose membrane by western blotting overnight. After blocking with 4% powdered milk in PBS for one hour, the membranes were incubated with rabbit anti-calreticulin antibody (Bio-techne, Abingdon, UK; NB600-103SS; 1:500) in dilution buffer (1% fetal bovine serum, 1% horse serum, 0.1% BSA in PBS with 0.05% Triton X-100) at room temperature for one hour, followed by incubation with HRP-labelled goat anti-rabbit Ig (Agilent, Stockport, UK; P0488) in dilution buffer at 0.25 ng/mL. Between each step membranes were washed three times with PBS for five minutes. Membranes were incubated with West Femto (Thermo Fisher, Paisley, UK) and visualized using a Gel Image Documentation system (Bio-Rad, Deeside, UK). Densitometry measurements of antibody reactive bands were obtained using Fiji software (version 1.51) [28] and were normalized to densitometry measurements of the Coomassie stained gel. For densitometry measurements only, contrast and brightness were adjusted uniformly across the gel and blot to decrease the background and enhance signal detection. After first testing whether the data was normally distributed using a Shapiro–Wilk normality test, independent a two-tail *t*-test was used to determine whether there was a statistically significant difference between WT and ALS mice, and a Mann–Whitney *U* test was used to test for significant difference between healthy controls and SMA mice. All statistical analyses were performed in SPSS version 24 (IBM, New York, NY, USA).

### 2.7. Immunohistochemistry

For immunohistochemistry, spinal cords from P8 SMA mice and healthy littermates, and 20 week-old ALS mice and WT littermates were fixed in 4% PFA for 24 h, followed by cryopreservation in 30% sucrose for another 24 h. Lumbar spinal cord sections (25 μm) were permeabilised with 0.3% Triton X-100 in PBS for ten minutes and blocked for one hour in 4% BSA/PBS with 0.1% Triton X-100 or in 10% goat serum/PBS. After incubation with rabbit anti-calreticulin antibody (Abcam, Cambridge, UK; EPR3924; 1:250) in blocking buffer (4% BSA/PBS with 0.1% Triton X-100 or 4% BSA/PBS with 0.1% Triton X-100 and 1% goat serum) overnight at 4 °C, sections were incubated with goat anti-rabbit IgG Alexa Fluor 546 (Life Technologies, Warrington, UK; A11010) or goat anti-rabbit IgG Alexa Fluor 488 (Life Technologies, Warrington, UK; A11034) for one hour at 5 μg/mL, followed by 4′,6-diamidino-2-phenylindole (DAPI) staining (Sigma Aldrich, Gillingham, UK; 0.4 μg/mL) for 10 min and mounted using Hydromount (Fisher Scientific, Loughborough, UK). Between each step sections were washed with PBS three times for ten minutes. Images were obtained using Leica SP5 confocal microscope with 63× oil immersion objective. Densitometry measurements of calreticulin staining in motor neurons of the anterior horn were performed using Fiji software (version 1.51) [28]. First, images were calibrated using a calibrated step tablet. A rectangular selection that filled most of the step without overlapping another one was created to measure mean grey value of the first step. The same rectangular selection was then used to measure the mean grey value of all the consecutive steps in the tablet. Measured values were plotted against standard measurements of optical density (OD) to create a calibration curve. Prior to analysis, the mean OD of the negative control was subtracted from the image to correct the background. Calreticulin staining in the anterior horn of lumbar spinal cord sections was assessed in two control and two SMA mice (P8), and in two WT and two ALS mice (20 weeks). A minimum of four sections from each mouse was used in the analysis. Fields of view were selected from each section, and measurements of three distinct regions that contained alpha motor neurons were taken from each field of view using a rectangular selection tool of fixed size. A minimum of 45 measurements was made from each experimental group. Statistical analysis was performed in SPSS version 24 (IBM, New York, NY, USA). The Shapiro-Wilk test was used to assess the distribution of quantitative data, followed by a Mann-Whitney *U* test to determine whether there was a statistically significant difference in calreticulin staining between control and SMA mice, and between WT and ALS mice.

### 2.8. Quantitative PCR

Spinal cords were harvested from P7 SMA mice and healthy littermates as well as from 20-week-old *SOD1^G93A^* males and age/gender matched WT littermates. RNA was extracted with the RNeasy MiniKit (Qiagen, Manchester, UK). Reverse transcription was performed using the High-Capacity cDNA Reverse Transcription Kit (Applied Biosystems, Loughborough, UK) for spinal cords from SMA and control mice. For ALS and WT mice, reverse transcription was performed using the qPCRBIO cDNA Synthesis Kit (PCR Biosystems, London, UK). qPCRs were performed using qPCRBIO SyGreen Blue Mix Hi-ROX (PCR Biosystems, London, UK). Primers were as follows: PolJ F: ACCACACTCTGGGGAACATC; PolJ R: CTCGCTGATGAGGTCTGTGA; Calreticulin F: AAGACTGGGATGAACGAGCCAAGA; Calreticulin R: AATTTGACGTGGTTTCCACTCGCC. RNA polymerase II polypeptide J (PolJ), was used as a validated housekeeping gene as it has previously been demonstrated as being stably expressed between tissues and in different pharmacological conditions [29]. Relative gene expression was quantified using the Pfaffl method [30] and primer efficiencies were calculated with the LinReg PCR software version 2012.0. Statistical analysis was performed in SPSS version 24 (IBM, New York, NY, USA). The Shapiro–Wilk test was used to test normality, followed by independent two-tail *t*-tests to determine whether there was a statistically significant difference between groups.

## 3. Results

### 3.1. Overview of ALS Proteomic Studies

A total of 33 proteomic studies of ALS were eligible for comparison (Table S1) [20,31,32,33,34,35,36,37,38,39,40,41,42,43,44,45,46,47,48,49,50,51,52,53,54,55,56,57,58,59,60,61,62]. As expected, the number of differentially expressed proteins identified in studies correlated with the sensitivity of the method used, with label-free technologies having identified by far the greatest number of protein changes compared to 2D-gel approaches. Both in vivo and in vitro models of ALS were used in analyses, and samples from animal models and ALS patients were equally represented across studies (Table S1). Samples were derived from a range of sources, including ALS patient cerebrospinal fluid (CSF) [20,31,32,33,34,35,36,37,38,39,40], patient serum [41], a range of cell models [42,44,57,59], ALS patient muscle [45,46], ALS patient blood mononuclear cells (PBMC) [48], ALS patient spinal cord [61], ALS patient prefrontal cortex [62], and tissues/cells from various mouse and rat models of ALS including astrocytes [47], skeletal muscle [53], ventral roots [50], embryonic motor neurons [51], spinal cord [43,49,52,54,55,56,58], and hippocampus [60].

### 3.2. Multi-Study Proteomic Identification of Conserved Molecular Response in ALS Patients and ALS Experimental Models

Protein expression changes in biofluids may not necessarily correlate with protein changes at the cellular level and may in fact be contradictory. It is possible that secretion or leakage of proteins into the CSF or serum may, for example, be accompanied by a concomitant decrease of expression levels in tissues [63]. Thus, studies that utilized cells/tissues were compared separately to those that examined biofluids. Comparison across the 12 proteomic studies that examined biofluids from ALS patients [20,31,32,33,34,35,36,37,38,39,40,41] identified 11 proteins that were consistently changed in the same direction across at least two studies (Table S2). Of these, one protein—cystatin C—was decreased in expression across four separate studies [35,37,38,39] and one protein—chitinase 3-like protein 1—was increased in expression across three separate studies [32,34,41]. The remaining eight proteins showed opposing directions of differential expression (Table S3). Gene ontology (GO) analysis was performed using the DAVID software [18,19] to investigate the likely function of the 11 proteins that showed a consistent change in expression. “Establishment of localization” was the most enriched biological process term, with seven of the eleven proteins mapping to it (Figure S1A), and complementary to this, 9 of the 11 proteins were associated with the term “vesicles”; eight of which were connected to the term “extracellular vesicles” (Figure S1B). STRING 10 analysis identified association network between four proteins: HBA1, HP, TF and ORM1 (Figure S1D), all of which appear to be plasma-derived proteins.

Comparison of the 21 studies that investigated proteome changes in tissues and cells from ALS patients and animal models identified 80 proteins that were differentially expressed across two or more studies. Of these, 42 proteins showed a consistent direction of differential expression (Table S4), eleven of which were upregulated across three or more studies: aldolase A [47,48,49,53,57], superoxide dismutase 1 [53,54,56] and 2 [44,48,52,58], 14-3-3 protein gamma [44,52,54], calreticulin [44,48,50], glyceraldehyde-3-phosphate dehydrogenase [49,53,54], heatshock protein 1 [44,54,56], heatshock protein family A member 8 [44,48,54], peroxiredoxin 2 [44,48,51] and 6 [48,49,56] and glial fibrillary acidic protein [47,54,62]. The remaining 38 proteins showed a contradictory direction of expression in different studies (Table S5). 

**Figure 1:**
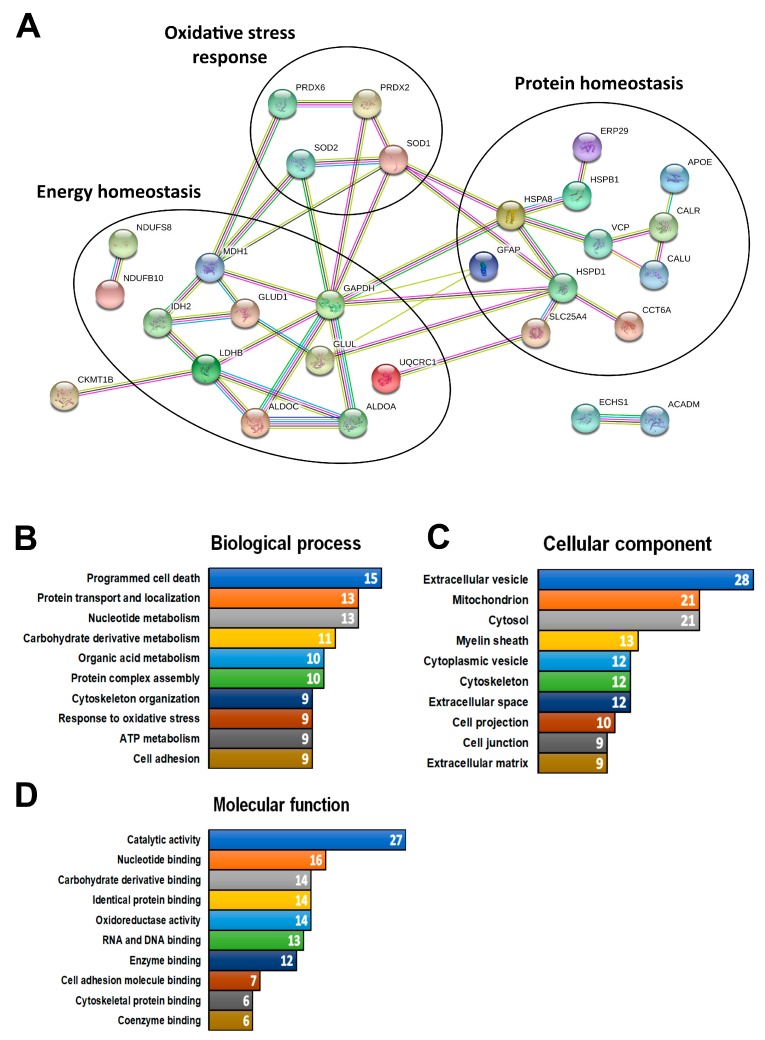
Bioinformatics analysis of the proteins consistently changed in the same direction in amyotrophic lateral sclerosis (ALS) tissues and cells. (**A**) Search Tool for the Retrieval of Interacting Genes/Proteins (STRING) 10 association network showing only the proteins that demonstrated association. The type of association between proteins is indicated by the color (pink: experimentally determined interactors; light blue: interactors form curated database; grey: protein homology; black: co-expression; yellow: text-mining; dark blue: gene co-occurrence; green: gene neighborhood). Three functional groups of proteins associated with regulation of oxidative stress, energy homeostasis and protein homeostasis were identified in the network from Figure 1B. Gene ontology (GO) term analysis using the DAVID platform revealed enriched terms connected to (**B**) biological processes, (**C**) cellular component and (**D**) molecular function. The top 10 terms from each domain were presented as bars. White numbers inside the bars indicated the number of annotated proteins. The full list of terms together with annotated proteins is available in Table S6.

STRING 10 analysis of the 42 proteins that showed a consistent change in expression revealed strong association between mitochondrial and endoplasmic reticulum proteins that are involved in the control of energy homeostasis, oxidative stress response and protein homeostasis (Figure 1A and Table S6). Within the networks, glyceraldehyde-3-phosphate dehydrogenase (GAPDH) was found to have the greatest number of known associations, linking with proteins involved in each of the three main domains. GO analysis of the 42 proteins using the DAVID platform supported these findings and highlighted enrichment of additional terms including programmed cell death, muscle tissue development and synaptic signaling (Figure 1B and Table S6). Cellular component GO analysis returned a range of terms, but similar to the bioinformatics finding from the CSF studies, the greatest number (i.e., 64%) of annotated proteins were associated with the term “extracellular vesicles” (Figure 1C). Enriched terms in the domain “molecular function” were predominantly associated with binding activity (Figure 1D). 

### 3.3. Multi-Study Proteomic Identification of Conserved Molecular Perturbations in both SMA and ALS

**Figure 2:**
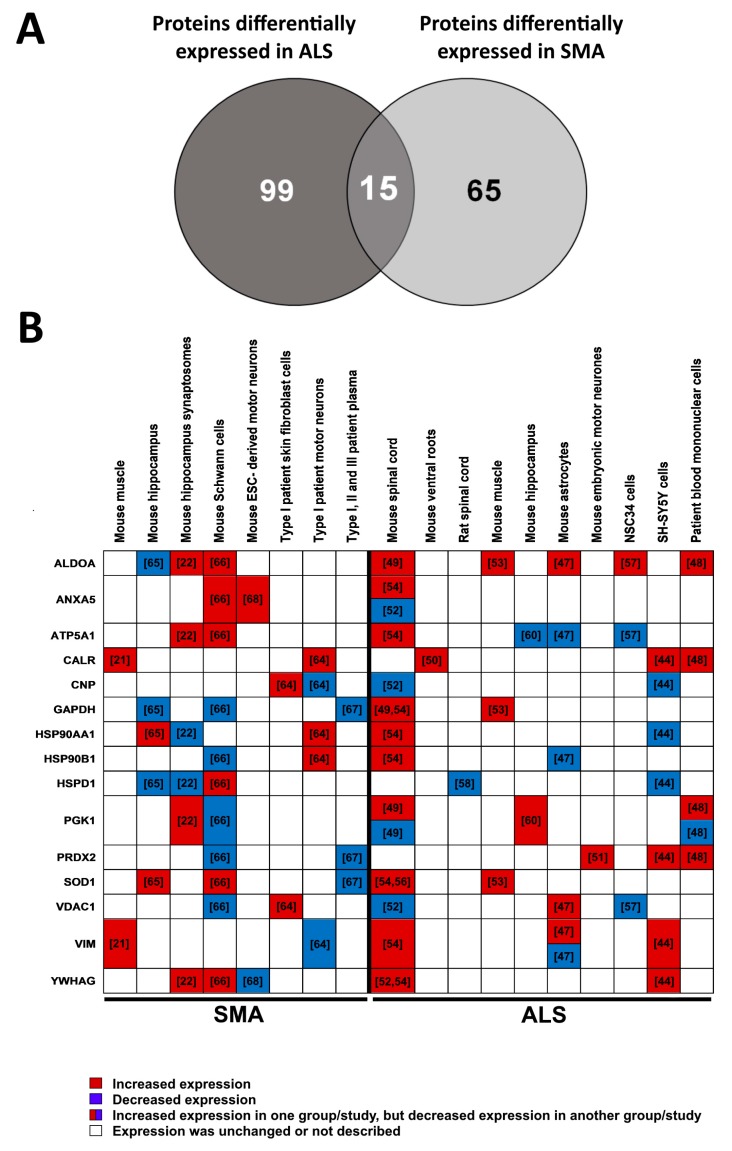
Proteins differentially expressed in both spinal muscular atrophy (SMA) and ALS proteomic studies. (**A**) Venn diagram showing the number of proteins differentially expressed in SMA proteomic studies compared to ALS proteomic studies. Fifteen of those proteins were identified in both SMA and ALS comparison. (**B**) Heat map of proteins that were differentially expressed in both SMA and ALS proteomic studies. Protein names are presented as official gene symbols. The reference for each study is given in the corresponding squares of the heat map. Experimental model and sample type used in each study are indicated above the table. ALDOA—Aldolase, Fructose-Bisphosphate A; ANXA5—Annexin A5; ATP5A1—ATP Synthase Subunit Alpha Mitochondrial; CALR—Calreticulin; CNP—2,3-Cyclic Nucleotide 3-Phosphodiesterase; GAPDH—Glyceraldehyde-3-Phosphate Dehydrogenase; HSP90AA1—Heat Shock Protein HSP 90 Alpha; HSP90B1—Heat Shock Protein 90 Beta Family Member 1; HSPD1—Heat Shock Protein Family D, Member 1; PGK1—Phosphoglycerate Kinase 1; PRDX2—Peroxiredoxin 2; SOD1—Superoxide Dismutase 1; VDAC1—Voltage Dependent Anion Channel 1; VIM—Vimentin; YWHAG—14-3-3 Protein Gamma.

To identify conserved protein changes common to SMA and ALS, differentially expressed proteins identified in the ALS multi-study comparison (Tables S2–S5) were compared with the differentially expressed proteins identified in our previous multi-study comparison of SMA proteomic datasets [16]. The following 15 proteins were found to be differentially expressed in both diseases and are summarised in Figure 2: calreticulin [21,44,48,50,64], superoxide dismutase 1 [53,54,56,65,66,67], aldolase fructose-bisphosphate A [22,47,48,49,53,57,65,66], heat shock protein family D, member 1 [22,44,58,65,66], peroxiredoxin 2 [44,48,51,66,67], voltage dependent anion channel 1 [47,52,57,64,66], vimentin [21,44,47,54,64], phosphoglycerate kinase 1 [22,48,49,60,66], annexin A5 [52,54,66,68], heat shock protein 90 beta family member 1 [47,54,64,66], glyceraldehyde-3-phosphate dehydrogenase [49,53,54,65,66,67], heat shock protein HSP 90 alpha [22,44,54,64,65], ATP synthase subunit alpha mitochondrial [22,47,54,57,60,66], 14-3-3 protein gamma [22,44,52,54,66,68], 2,3-cyclic nucleotide 3-phosphodiesterase [44,52,64].

**Figure 3:**
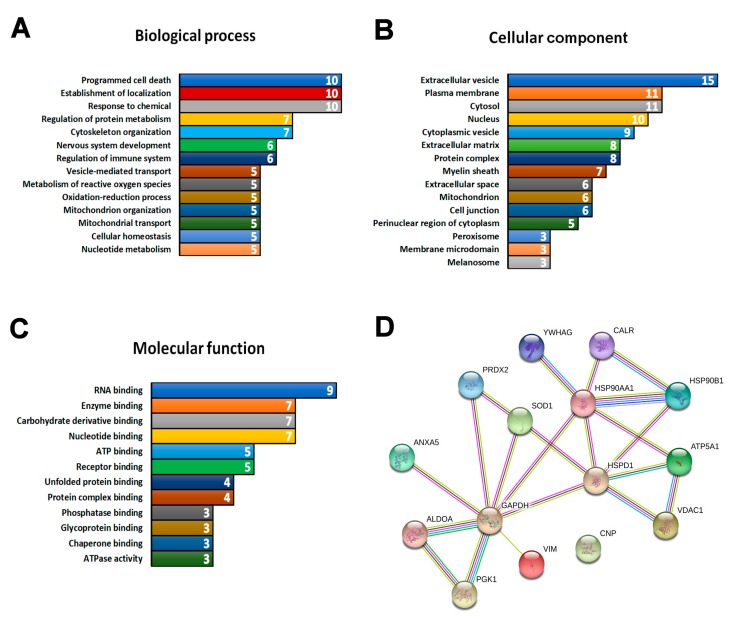
Bioinformatics analysis of the 15 proteins differentially expressed in both SMA and ALS proteomic studies. Gene ontology analysis revealed enriched terms connected to (**A**) biological process, (**B**) cellular component, and (**C**) molecular function. In the biological process domain, only terms with five or more annotated proteins are presented. Terms are presented as bars, with the white numbers inside the bars indicating number of annotated proteins. The full list of terms together with annotated proteins is available in Table S7. (**D**) STRING 10 association network of proteins that were differentially expressed in SMA and ALS. Proteins connected to metabolic function, oxidative stress response and protein stability were identified in the network from Figure 3A. The type of the association between proteins is indicated by the color (pink: experimentally determined interactors; light blue: interactors form curated databases; grey: protein homology; black: co-expression; yellow: text-mining; dark blue: gene co-occurrence; green: gene neighborhood).

Programmed cell death was one of the most enriched biological process GO terms (Figure 3A) and is likely describing the downstream consequences of disease pathogenesis. Other highly enriched terms included regulation of immune system, regulation of protein metabolism, cytoskeleton organization, and the metabolism of reactive oxygen species. Strikingly, all 15 proteins identified in the comparison were associated with the cellular component term “extracellular vesicles” (Figure 3B). Transport and establishment of localization were also among enriched biological process GO terms (Figure 3A). Enriched terms in the domain “molecular function” were associated with binding activity (Figure 3C). STRING 10 analyses revealed a strong association network between 14 of the 15 proteins (Figure 3D).

**Figure 4:**
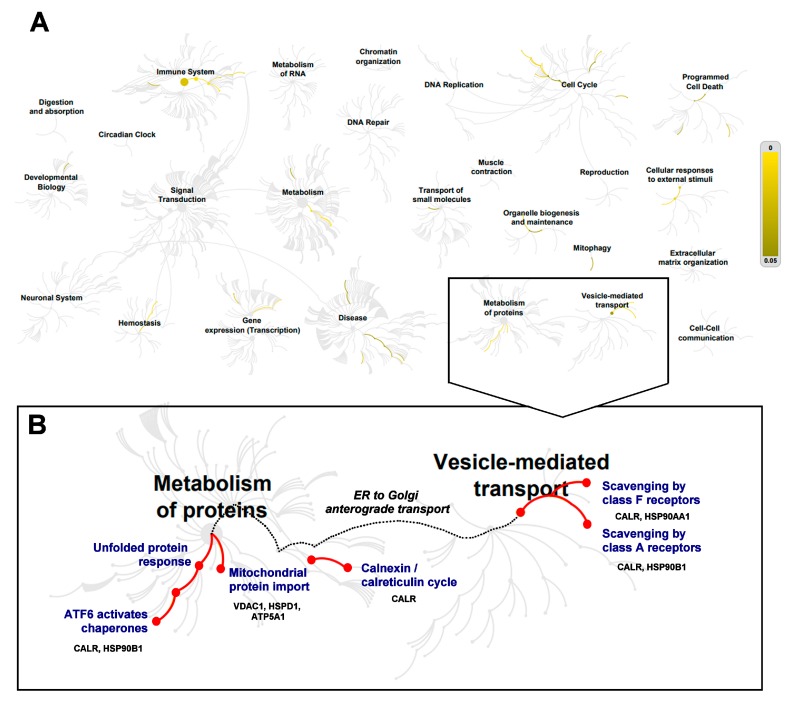
Reactome analysis of the 15 proteins differentially expressed in both SMA and ALS proteomic studies. (**A**) Genome-wide overview of Reactome pathway analysis identified several over-represented pathways (highlighted using a colored scale on the right-hand side that indicates false discovery rate (FDR)), including endoplasmic reticulum (ER) to Golgi anterograde transport [69], (**B**) Zoomed view of “metabolism of proteins” and “vesicle-mediated transport” pathways. Over-represented pathways are indicated by the red line. Proteins annotated to each pathway are shown.

Reactome pathway analysis [24] was performed to further explore which pathways the 15 proteins dysregulated in both ALS and SMA are associated with. At the genome-wide view, pathways that were over-represented were largely complementary to those identified by the GO analysis, above (Figure 4A). Of the over-represented pathways identified, only two—“metabolism of proteins” and “vesicle-mediated transport”—were shown to have a direct connection to each other (Figure 4B) [69]. The molecular highway connecting these two pathways is the “endoplasmic reticulum (ER) to Golgi anterograde transport” pathway [69], suggesting this as a core pathway upon which ALS and SMA disease mechanisms converge. Of particular note is that calreticulin (CALR) was associated with both “metabolism of proteins” and “vesicle-mediated transport” pathways (Figure 4B) and was the only protein identified with a consistent direction of differential expression across SMA and ALS proteomic studies (Figure 2B).

### 3.4. Calreticulin Expression is Dysregulated in Spinal Cord Tissue from ALS and SMA Mice

**Figure 5:**
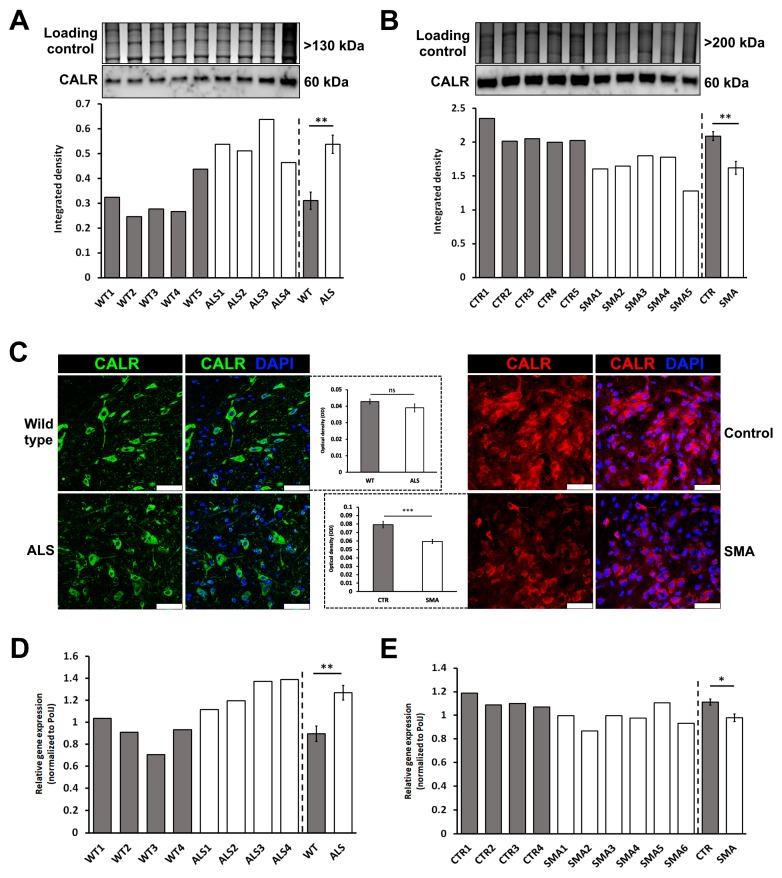
Dysregulated expression of calreticulin in spinal cord extracts from late-symptomatic ALS mice (20 week) and late-symptomatic SMA mice (P8). Representative western blots showing (**A**) a 43% increase in calreticulin (CALR) expression levels in spinal cords of ALS mice (*n* = 4) compared to wild type (WT) mice (*n* = 5); (**B**) a 26% reduction of calreticulin expression in SMA mice (*n* = 5) compared to control mice (*n* = 5). Graphs are presented as integrated density of measured protein normalized to total protein (Coomassie stained gel). Densitometry measurements for individual samples and mean of the group are shown, with error bars showing standard error from the mean; (**C**) Representative immunohistochemistry images showing calreticulin expression in the ventral horn of lumbar spinal cord sections in 20-week-old WT and ALS mice and in control and age-matched (P8) SMA mice. Scale bar = 50 μm. Densitometry measurements of calreticulin levels in alpha motor neurons are presented as mean optical density, with error bars showing standard error from the mean. (**D**) *Calreticulin* gene expression levels in WT and ALS mice, and; (**E**) in control and SMA mice, as determined by RT-PCR. Expression levels of *calreticulin* were normalized to *PolJ*. Error bars represent standard error from the mean. CALR—Calreticulin; CTR—Control; WT—Wild Type. ns—Not Significant; * *p* < 0.05; ** *p* < 0.01; *** *p* < 0.001.

None of the five proteomic studies that analyzed whole spinal cord extracts from mouse or rat models of ALS reported detection of calreticulin [49,52,54,55,56] (Table S1) (but this is perhaps not surprising since each of these studies utilized a 2D-gel electrophoresis protocol which identifies significantly fewer proteins compared to more recent techniques such as iTRAQ or label-free quantification). Though induced pluripotent stem cell (iPSC)-derived motor neurons have been studied in the context of SMA [64], there have been no proteomic studies of spinal cord or primary motor neurons isolated from animal models of SMA to date. We therefore performed quantitative western blot analysis of spinal cord extracts from mouse models of ALS (*SOD1^G93A^*) and SMA (severe ‘Taiwanese’ model) at late symptomatic time-points to determine the expression levels of calreticulin relative to healthy littermate controls. In spinal cord extracts from ALS mice, calreticulin expression was significantly increased by an average of 43% (*p* = 0.003) (Figure 5A). In contrast, however, calreticulin expression in SMA spinal cord extracts was significantly reduced by 22% compared to that of controls (*p* = 0.008) (Figure 5B). Immunohistochemistry analysis of the ventral horn of lumbar spinal cord sections from control and age-matched (P8) SMA mice closely aligned with the quantitative western blotting, showing calreticulin was reduced by an average of 26% in SMA compared to controls (*p* < 0.001) (Figure 5C). A significant difference in calreticulin expression was not detected by immunohistochemistry analysis of ALS and WT lumbar spinal cord sections (Figure 5C), but RT-PCR analysis of *calreticulin* gene expression aligned with the trends seen by western blot results, showing a statistically significant increase in ALS vs. WT (Figure 5D) and a slight, but statistically significant, decrease in SMA vs. control spinal cords (Figure 5E).

## 4. Discussion

Here, we used a comparative approach to identify core molecular perturbations shared between SMA and ALS with the aim of understanding common molecular mechanisms of disease pathogenesis. Multi-study comparisons of published proteomic datasets identified 15 proteins that were dysregulated across two of more separate studies in both SMA and ALS. These proteins therefore represent a potential “molecular fingerprint” of disease pathogenesis that is shared between both diseases. Bioinformatics analysis of the 15 differentially expressed proteins identified over-representation of several pathways including cell death, protein metabolism and vesicle-mediated transport pathways; with the latter two being linked via the ER to Golgi anterograde transport pathway. One of the 15 proteins, calreticulin, was strongly associated with this pathway because of its involvement with both protein metabolism and vesicle-mediated transport pathways [69,70]. Using quantitative western blot and immunohistochemistry, we demonstrated for the first time that calreticulin is reduced in expression in spinal cords from late-symptomatic SMA mice, and in agreement with proteomic studies of other ALS cells and tissue [44,48,50], we measured increased levels of calreticulin in spinal cord extracts from symptomatic ALS *SOD1^G93A^* mice. 

Golgi fragmentation and ER stress were both identified as key pathological features in sALS [71] and transgenic mouse models of fALS [72], and both were evident at a pre-symptomatic stage of disease, directly implicating them in disease pathogenesis [72,73]. It was only recently, however, by studying induced pluripotent derived-motor neurons from SMA patients, that ER stress was implicated in SMA pathogenesis [74]. In line with this, ER stress was also found to be a prominent feature of spinal motor neurons from a mouse model of X-linked spinal and bulbar muscular atrophy [75], and alterations of Golgi apparatus were previously reported in anterior horn cells from patients with X-linked spinal and bulbar muscular atrophy [76] and in fibroblasts from patients with a rare autosomal dominant form of SMA [77]. 

ER stress and Golgi alterations can both be triggered by impaired ER-Golgi transport mechanisms, ultimately leading to induction of the cell death-related pathways [78,79]. Dysregulated protein transport between ER and Golgi, identified in the bioinformatics analysis here, therefore highlights the possibility that the same biological mechanism is responsible for aspects of disease pathogenesis in SMA and ALS. Indeed, ALS-associated mutant SOD1, TDP-43 and FUS proteins have been reported to inhibit ER-Golgi trafficking in neuronal cells [78,80]. Importantly, dysregulation of ER-Golgi trafficking preceded protein aggregation, ER stress, Golgi fragmentation and axon degeneration in NSC-34 cells expressing mutant SOD1 [78], and dysregulated ER-Golgi trafficking was observed as an early event in embryonic cortical and motor neurons in a mouse model of ALS [80]. Over-expression of *Rab1*, a master regulator of ER-Golgi transport, restored ER-Golgi trafficking and prevented induction of ER stress, formation of intracellular aggregations and apoptosis in in vitro model of ALS [80]. Several studies also indicate a role for defective ER-Golgi transport in SMA. SMN interacts with *α-COP*, a member of COPI vesicles that mediates ER-Golgi transport [81,82,83,84], and knockdown of *α-COP* caused SMN accumulation in Golgi apparatus of neuron-like NSC34 cells [82] and produced developmental defects in motor neuron-like NSC34 cells and primary cortical murine neurons [84]. Over-expression of human *α-COP* reversed motor neuron defects in SMN-depleted NSC34 cells [83,84] and in motor neurons from a zebrafish model of SMA [84]. A role for defective ER to Golgi transport in ALS and SMA pathogenesis is further supported by our finding here that all 15 of the differentially expressed proteins, common to SMA and ALS, mapped to “extracellular vesicles” in cellular compartment GO analysis.

Calreticulin is a multifunctional protein involved in a range of processes, including the regulation of Ca^2+^ homeostasis in the ER, chaperone activity in secretory pathways, folding of nascent proteins, regulation of immune system, and modulation of cell adhesion [17]. Its chaperone activity in the ER is dependent on calcium (Ca^2+^) levels and perturbations in Ca^2+^ homeostasis can disrupt the correct functioning of protein folding machinery [85]. It has been suggested that defective Ca^2+^ handling is responsible for triggering calreticulin depletion in ALS vulnerable motor neurons, leading to ER stress and apoptosis [86]. In addition, defects in Ca^2+^ handling have been demonstrated in both SMA [87,88,89] and ALS [90,91,92]. Alterations in calcium homeostasis are also known to inhibit ER-Golgi trafficking [93], indicating a possible common mechanism that drives ER and Golgi defects in SMA and ALS. Calreticulin was previously identified from proteomic studies as having increased levels in iPSC-derived motor neurons from type I SMA patients [64] and in SMA mouse muscle [21], and this was verified biochemically in muscles from SMA mice and SMA patients [21]. In contrast, we observed a statistically significant decrease of calreticulin levels in spinal cord extracts from late symptomatic mice. In proteomic studies of ALS, calreticulin was found to be increased in the ventral roots from the spinal cord of ALS *SOD1^G93A^* mice [50], blood mononuclear cells from ALS patients [48], and in TDP-43 knockdown SH-SY5Y cells [44], and in agreement with this, we also found increased levels of calreticulin in spinal cord extracts from late-symptomatic ALS *SOD1^G93A^* mice. This is, however, in contrast with a biochemical study of lower motor neurons isolated from ALS *SOD1^G93A^* mice that reported decreased expression of calreticulin [86]. 

When taken together, there is certainly evidence to support a role for calreticulin dysregulation in both ALS and SMA, but it is important too, to consider possible explanations for the opposing patterns of calreticulin expression reported in different studies, as this may offer further insights into disease mechanisms. In the case of whole spinal cord from ALS mice, for example, differences in calreticulin expression in different cell populations could, according to a theory proposed by Bernard-Marissal et al. [86], have contributed to the overall calreticulin expression levels, masking changes that were previously reported to be specific to vulnerable lower motor neurons [86]. This could also offer an explanation for why western blotting and RT-PCR analysis of whole spinal cord extracts revealed a statistically significant increase in calreticulin protein and gene expression in ALS, but immunohistochemistry analysis of lumbar spinal cord sections did not. It has been shown that calreticulin levels increase under stress conditions prior to being secreted to the cell surface; a process that is associated with the functional role of calreticulin in apoptosis [94]. It is thus possible that opposing directions of perturbed calreticulin expression reflect ongoing cycles of apoptosis in vulnerable motor neurons. The time-course of disease is another potential variable to consider, since Bernard-Marissal et al. [86] studied ALS mice up to 110 days of age, compared to the later time-point of 140 days in the present study. The obvious limitations of using a late-symptomatic animal model are that it cannot determine whether changes in protein expression levels are causative of disease pathology or are simply reflective of cell death processes. It would be of interest, therefore, to verify the expression of calreticulin throughout different stages of disease development in both SMA and ALS and determine whether other cell populations in proximity of vulnerable motor neurons or unaffected spinal cord regions contribute to alterations in calreticulin levels. Further work is clearly warranted too, to examine the relationship between calreticulin perturbations, defective Ca^2+^ handling, and ER and Golgi defects in SMA and ALS, and whether targeting these pathways offers a potential therapeutic approach to both diseases.

In this study we demonstrated that calreticulin expression is altered at both the transcript and protein level in ALS and SMA, so we were interested to determine the extent to which the other proteomic changes common to ALS and SMA can be traced back to aberrant gene regulation. None of the SMA and ALS proteomic studies used in this comparison also investigated changes using transcriptomics approaches, but a small number of transcriptomic studies of SMA and ALS have been conducted separately using comparable experimental models. For ALS, two transcriptomic studies investigated gene alterations in patient blood mononuclear cells [95,96], but none of the four proteins found to be differentially expressed in the proteomic study (Table S2) were reported to be differentially expressed at the gene level in either of the two studies. One of the eight proteins that were differentially expressed in spinal cords from ALS *SOD1^G93A^* mice [54] (Figure 2), ATP5A1, was also found to be differentially expressed at the gene level in a separate study [97]; with both proteomic and transcriptomic studies having reported an increased expression in ALS. For SMA, two proteins were differentially expressed in mouse embryonic stem cell (ESC)-derived motor neurons (Figure 2) [68], and one of these—ANXA5—was also found to be differentially expressed in a separate transcriptomic study [98], but with the opposite direction of change. None of the five proteins differentially expressed in iPSC-derived motor neurons from Type I SMA patients (Figure 2) [64] were found to be differentially expressed in a transcriptomic study of similar cell lines [74]. The altered expression of calreticulin (verified in this study at both transcript and protein level), and the altered expression of ATP5A1 at the protein level (Figure 2) [22,47,54,57,60,66] and at the transcript level [97], suggest that these molecular changes could be due to aberrant gene transcription. The majority of well-characterized molecular changes in SMA and in many fALS cases, however, have been traditionally attributed to dysregulation of RNA metabolism [14], and the apparent lack of overlap between the profile of differentially expressed proteins summarised in this study with differentially expressed genes found in separate studies supports the possibility that at least some changes may be due to post-transcriptional gene regulation defects. Perturbed post-transcriptional gene regulation in both diseases has been associated with several defective RNA processing pathways, including those that regulate RNA splicing [99], RNA stability and translation [100,101]. A growing body of evidence also suggests physical and functional links between SMA and ALS-associated proteins, including SMN, FUS, TDP43 and SOD1, some of which are RNA-binding proteins (summarized in recent reviews [14,15]). This may also offer an explanation of how perturbations in RNA processing pathways extend to fALS cases with mutations in non-RNA binding proteins such as SOD1 [14]. Taken together, it seems that aberrant post-transcriptional gene regulation might contribute to some, but perhaps not all, of the molecular defects in SMA and ALS. Further work is clearly warranted though to establish the precise relationship between ALS and SMA transcriptional and post-transcriptional changes and protein expression changes. When doing so, it will be important to consider these relationships in the same cell/tissue type, matched for both relative age and disease timeline. 

The aim of this study was to identify the molecular overlap between SMA and ALS, but it is important not to overlook the results from the comparative analysis of ALS proteomic data sets (Tables S2–S5) as these are likely to provide a useful resource for research aimed at understanding ALS-specific pathways and biomarkers. Whilst several of these proteins have gained significant attention in the context of ALS, including the putative auxiliary biomarker, cystatin C [102] and SOD1 [15], several proteins appear to have been overlooked when these data sets were considered in isolation. One such protein, aldolase A, for example, was increased in expression in five separate studies of ALS cells and tissues [47,48,49,53,57]. Given its importance in glycolysis [103], overexpression of this protein is highly likely to reflect perturbed metabolic activity. Indeed, mitochondrial dysfunction [104] and ER stress [105] are known early pathological features and a major contributing factor of motor neuron death in ALS. Not surprisingly, most of the dysregulated proteins identified in tissues and cells from ALS patients and models were associated with energy homeostasis, oxidative stress response and control of protein stability, confirming the importance of ER and mitochondria in ALS disease pathways. Recently, defective ER-mitochondria crosstalk has received attention in the context of ALS pathogenesis, implying that ER and mitochondria perturbations cannot be perceived separately, but rather as a complex network of changes that together contribute to ALS pathogenesis [106,107]. This therefore presents an opportunity for the development of therapeutic approaches for ALS to simultaneously target functional defects in the ER and mitochondria [106,107].

Another important finding is that most proteins with a consistent direction of differential expression across ALS proteomic studies were associated with extracellular vesicles. Significant attention has been given to extracellular vesicles in the context of neurodegenerative disorders, including ALS, as a potential source of biomarkers and as a possible mechanism of misfolded protein spreading in CNS [108]. Mutant SOD1 and TDP-43 proteins are among molecules whose prion-like properties, facilitated by exosome transport, are thought to contribute to spreading of protein misfolding in ALS [108]. Interestingly, all 15 proteins identified in both ALS and SMA were also associated with extracellular vesicles. A recent study reported increased levels of exosomes in culture media from SMN-depleted cells, SMA patient fibroblasts, and in serum from SMA patients and mouse model of SMA [109], indicating alterations in exosome transport as another shared link between SMA and ALS.

To conclude, we provide evidence that integrating multi-study comparisons of proteomic datasets with bioinformatics analysis can help to unravel complex disease mechanisms. The 15 proteins identified here represent the core molecular overlap between SMA and ALS; many of which, have not until now, been considered in isolation. The identification of over-represented pathways, including two that converge on ER-Golgi trafficking, offers further insights into mechanistic cross-talk between SMA and ALS. Together, these findings offer significant insights into potential common targets that may help to guide the development of new therapies for both diseases.

## Supplementary Materials

The following are available online at https://www.mdpi.com/2076-3425/8/12/212/s1, Table S1: ALS proteomic studies used in the comparison, Table S2. Proteins differentially expressed in the same direction in biofluids from ALS patients, Table S3. Proteins that showed contradictory direction of differential expression in biofluids from ALS patients across at least two proteomic studies, Figure S1. Bioinformatics analysis of eleven proteins that showed consistent change in expression in biofluids from ALS patients, Table S4. Proteins that showed consistent direction of differential expression in ALS cells and tissues across two or more proteomic studies, Table S5. Proteins that showed contradictory direction of differential expression in cells and tissues across two or more proteomic studies of ALS, Table S6. GO term analysis of proteins that were differentially expressed in tissues and cells in ALS proteomic studies, Table S7. GO term analysis of proteins that were differentially expressed in SMA and ALS proteomic studies.
