# Supplementary material for: Multi-Study Proteomic and Bioinformatic Identification of Molecular Overlap between Amyotrophic Lateral Sclerosis (ALS) and Spinal Muscular Atrophy (SMA)
Source: Brain Sci. 2018 Dec 4;8(12):212. doi: 10.3390/brainsci8120212 (PMC6315439; doi:10.3390/brainsci8120212)
Supplement: Supplementary file 1 [file brainsci-08-00212-s001.pdf]

brain sciences

MDPI

Table S1. ALS proteomic studies used in the comparison.

Ref	ALS Model/Sample Type	Differentially Expressed Proteins	Proteins included in Comparison	Analysis Platform	Protein Database
[20]	Patient CSF	35	35	iTRAQ; API QSTAR XL (Applied Biosystems)	UniProt/Swiss-Prot
[31]	Patient CSF	123	110	Label-Free; linear ion trap (ThermoFisher Scientific)	UniProt/Swiss-Prot
[32]	Patient CSF	48	47	iTRAQ; LTQ-Orbitrap Velos (Thermo Electron)	Human Protein Database (NCBI)
[33]	Patient CSF	17 *	17	2D electrophoresis; Voyager-DE Pro MALDI-TOF (Applied Biosystems)	NCBI and UniProt/Swiss-Prot
[34]	Patient CSF	153	11	Peak recognition (statistical analysis); linear Microflex MALDI-TOF mass spectrometer (Bruker)	Entrez Protein Database
[35]	Patient CSF	33	7	SELDI-TOF-MS	Empirical Proteomics Ontology Knowledge Base
[36]	Patient CSF	6	5	2D-DIGE; Voyager DE-STR (Applied Biosystems)	NCBI database
[37]	Patient CSF	52 †	3	SELDI-TOF-MS/MS; Ciphergen ProteinChip Reader (Ciphergen Biosystems)	UniProt/Swiss-Prot
[38]	Patient CSF	10 (PM-ALS) †<br>9 (L-ALS) †	2	SELDI-TOF-MS; Ciphergen ProteinChip Reader (Ciphergen Biosystems)	UniProt/Swiss-Prot
[39]	Patient CSF	3	2	SELDI-TOF-MS	Unknown
[40]	Patient CSF	3	3	UHPLC LC-MS/MS; Q Exactive HF tandem mass spectrometer (Thermo Fisher Scientific)	UniProt
[41]	Patient serum	7	7	2D-GE; SYNAPT-MS G1 (Waters Corporation)	UniProt
[42]	Rat neurons/TDP-43 Knockdown	63	63	Label-free; Orbitrap Q Exactive (Thermo Fisher Scientific)	UniProt/Swiss-Prot
[43]	Patient CSF injected into rat spinal cord /mitochondrial fraction	49	48	iTRAQ; LTQ-Orbitrap Velos (Thermo Electron)	RefSeq
[44]	SH-SY5Y cells/TDP-43 Loss	273	270	Label-free; LTQ Orbitrap XL (Thermo Fisher Scientific)	UniProt/Swiss-Prot
[45]	Patient muscle	5	5	2D-GE; API QStar PULSAR (AB-Sciex)	UniProt/Swiss-Prot
[46]	Patient muscle	11	11	Stable-isotope dimethyl labels; 7 T hybrid LTQ FT Ultra mass spectrometer (ThermoFisher Scientific)	UniProt/Swiss-Prot
[47]	Mouse astrocytes/SOD1 G93A	31	31	2D-GE; Reflex III MALDI-TOF (Bruker Daltonics)	UniProt/Swiss-Prot

[48]	Patient blood mononuclear cells	44	44	2D DIGE; 4800 MALDI TOF/TOF (Applied Biosystems)	UniProt/Swiss-Prot
[49]	Mouse spinal cord/Wobbler Mouse	13	13	2D DIGE; Model 6430 Ion Trap (Agilent Technologies)	NCBI
[50]	Mouse ventral roots/SOD1 G93A	14 #	14	Label Free; LTQ (Thermo Finnigan)	UniProt/Swiss-Prot
[51]	Mouse embryonic motor neurons/SOD1 G85R	6	4	2D-GE; UltraFlex MALDI TOF/TOF (Bruker-Franzen Analytik)	UniProt/Swiss-Prot
[52]	Mouse spinal cord/SOD1 G127X	53	47	2D-DIGE; Voyager DE-STR MALDI-TOF (Applied Biosystems)	UniProt/Swiss-Prot
[53]	Mouse muscle/Wobbler Mouse	31	26	2D-GE; Reflex III MALDI-TOF (Bruker Daltonics)	UniProt/Swiss-Prot
[54]	Mouse spinal cord (insoluble fraction)/SOD1 G93A	32	32	2DE; Reflex III MALDI-TOF (Bruker Daltonics)	UniProt/Swiss-Prot
[55]	Mouse spinal cord (pre-symptomatic)/SOD1 G93A	15	15	2D-GE; ReflexIII MALDI mass spectrometer (Bruker Daltonics)	UniProt/Swiss-Prot
[56]	Mouse spinal cord/SOD1 G93A	7	7	2D-GE; VG 2E Tofspec laser desorption time of flight mass spectrometer (Waters)	NCBI
[57]	NSC34 cells (mitochondrial fraction)/SOD1 G93A	40	38	2D-GE; Qstar XL Q-TO, Applied Biosystems	NCBI and UniProt/Swiss-Prot
[58]	Rat spinal cord (mitochondrial fraction)/SOD1 G93A	40	40	2D-GE; MudPIT RCADiA platform	Saccharomyces Genome Database/ Ensembl
[59]	NSC34 cells/SOD1 G93A/G37R	7	7	2D-GE; Voyager-DE STR, Perspective Biosystems	UniProt/Swiss-Prot
[60]	Mouse hippocampus/Tg152 line overexpressing hSOD1	41	41	2D-GE; Ultraflex MALDI TOF/TOF (Bruker Daltonics)	NCBI and UniProt/Swiss-Prot
[61]	Patient anterior and posterior horn	32 (anterior horn) 3 (posterior horn)	18 (anterior horn) 3 (posterior horn)	2D-GE; LTQ linear ion trap mass spectrometer (ThermoFisher Scientific)	UniProt
[62]	Patient prefrontal cortex	103	101	Label-free; Fusion mass spectrometer (ThermoFisher Scientific)	UniProt

ALS studies included in the proteomic comparison are listed in table, together with ALS model, sample type, analysis platform and database used in each study. Number of differentially expressed proteins identified in each study and number of those proteins that were included in the comparison are shown in column three and four. * Proteins were considered significantly changed if they were identified in ALS samples, but not in CTR samples. † Significantly changed proteins in ALS were identified by applying second-pass peak selection with a signal to noise ratio of 1.5 and $p < 0.01$ [8,9]. # By dynamically adjusting the *p*-value cut-off, *p*-value of 0.05 was chosen for the identification of significantly changed proteins in ALS. PM-ALS—Samples from ALS patients taken post-mortem; L-ALS—Samples from living patients; CSF—Cerebrospinal Fluid.

Table S2. Proteins differentially expressed in the same direction in biofluids from ALS patients.

Protein Name (Official Gene Symbol)	No. of Studies	Direction of Expression Change	Refs
Cystatin C (CST3)	4	Down	[35,37–39]
Alpha-1-Acid Glycoprotein 1 (ORM1)	2	Down	[20,32]
Apolipoprotein A-IV (APOA4)	2	Down	[34,41]
Haptoglobin (HP)	2	Down	[20,32]
Neurosecretory Protein VGF (VGF)	2	Down	[34,39]
Ribonuclease Pancreatic (RNASE1)	2	Down	[20,35]
Transferrin (TF)	2	Down	[32,36]
Chitinase 3-Like Protein 1 (CHI3L1)	3	Up	[32,34,41]
Chitinase 3-Like Protein 2 (CHI3L2)	2	Up	[31,32]
Chitotriosidase-1 (CHIT1)	2	Up	[32,41]
Hemoglobin Subunit Alpha 1 (HBA1)	2	Up	[20,35]

Proteins with a consistent change in expression in biofluids from ALS patients across two or more proteomic studies are shown. The protein name is given, followed by the official gene symbol in brackets.

Table S3. Proteins that showed contradictory direction of differential expression in biofluids from ALS patients across at least two proteomic studies.

Protein Name (Official Gene Symbol)	No. of Studies	Direction of Differential Expression	Refs
Transthyretin (TTR)	4	Down	[37,38,41]
		Up	[35] *
Hemoglobin subunit beta (HBB)	3	Up	[20,35]
		Down	[32]
Protein AMBP (AMBP)	3	Down	[20,34]
		Up	[41]
Apolipoprotein A-II (APOA2)	2	Down	[41]
		Up	[20]
Apolipoprotein B-100 (APOB)	2	Down	[31]
		Up	[32]
Prothrombin (F2)	2	Down	[34]
		Up	[32]
Testican-2 (SPOCK2)	2	Down	[31]
		Up	[20]
Zinc-Alpha-2-Glycoprotein (AZGP1)	2	Down	[20]
		Up	[36]

Proteins that showed contradictory change in expression in biofluids from ALS patients in two or more proteomic studies. Protein name is given, followed by official gene symbol in brackets. Direction of differential expression and number of studies that identified protein changes are shown (studies are listed in the reference column). * CysGly-transthyretin—Modified form of transthyretin generated by oxidative damage.

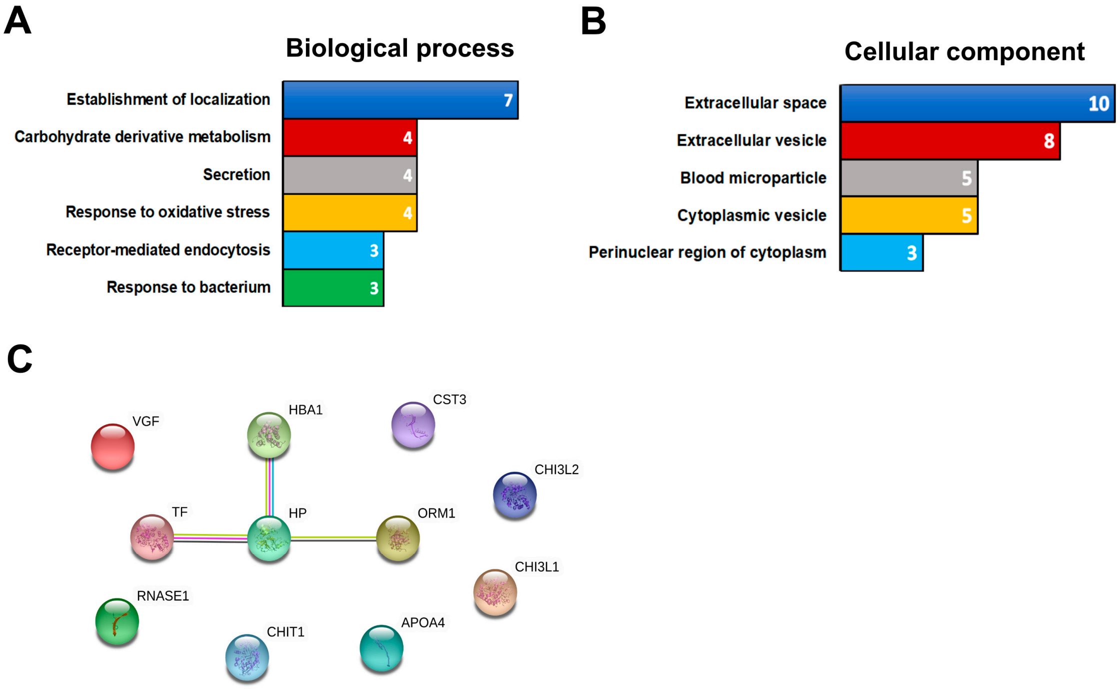

Figure S1. Bioinformatics analysis of eleven proteins that showed consistent change in expression across biofluids from ALS patients. Gene ontology analysis revealed enriched terms connected to (**A**) biological process, (**B**) cellular component. Terms are presented as bars, with the white numbers inside the bars indicating number of annotated proteins. (**C**) Search Tool for the Retrieval of Interacting Genes/Proteins (STRING) 10 analysis identified association network between four proteins: HBA1—Haemoglobin Subunit Alpha 1, HP—Haptoglobin, ORM1—Alpha-1-Acid Glycoprotein 1 and TF-transferrin. Type of the association between proteins is indicated by the colour (pink: experimentally determined interactors; light blue: interactors form curated databases; black: c89o-expression; yellow: text-mining).

Table S4. Proteins that showed consistent direction of differential expression in ALS cells and tissues across two or more proteomic studies.

Protein (Official Gene Symbol)	No. of Studies	Refs	Sample Type	ALS Model
Increased expression				
Aldolase A (ALDOA)	5	[57]	NSC34 cells (mitochondrial fraction)	SOD1 G93A
		[53]	Mouse muscle	Wobbler mouse
		[49]	Mouse spinal cord	Wobbler mouse
		[48]	Patient blood mononuclear cells	NA
		[47]	Mouse astrocytes	SOD1 G93A
Superoxide Dismutase 2 (SOD2)	4	[52]	Mouse spinal cord	SOD1 G127X
		[58]	Rat spinal cord (mitochondrial fraction)	SOD1 G93A
		[48]	Patient blood mononuclear cells	NA
		[44]	SH-SY5Y cells	TDP-43 Loss
Superoxide Dismutase 1 (SOD1)	3	[56]	Mouse spinal cord	SOD1 G93A
		[54]	Mouse spinal cord	SOD1 G93A
		[53]	Mouse muscle	Wobbler mouse
Peroxiredoxin 2 (PRDX2)	3	[51]	Mouse embryonic motor neurones	SOD1 G85R
		[48]	Patient blood mononuclear cells	NA
		[44]	SH-SY5Y cells	TDP-43 Loss
14-3-3 Protein Gamma (YWHAG)	3	[54]	Mouse spinal cord	SOD1 G93A
		[52]	Mouse spinal cord	SOD1 G127X
		[44]	SH-SY5Y cells	TDP-43 Loss
Heat Shock Protein 1 (HSPB1)	3	[56]	Mouse spinal cord	SOD1 G93A
		[54]	Mouse spinal cord	SOD1 G93A
		[44]	SH-SY5Y cells	TDP-43 Loss
Calreticulin (CALR)	3	[50]	Mouse ventral roots	SOD1 G93A
		[48]	Patient blood mononuclear cells	NA
		[44]	SH-SY5Y cells	TDP-43 Loss
Heat Shock Protein Family A, Member 8 (HSPA8)	3	[54]	Mouse spinal cord	SOD1 G93A
		[48]	Patient blood mononuclear cells	NA
		[44]	SH-SY5Y cells	TDP-43 Loss
Peroxiredoxin 6 (PRDX6)	3	[56]	Mouse spinal cord	SOD1 G93A
		[48]	Patient blood mononuclear cells	NA

		[49]	Mouse spinal cord	Wobbler mouse
Glial Fibrillary Acidic Protein (GFAP)	3	[54]	Mouse spinal cord	SOD1 G93A
		[47]	Mouse astrocytes	SOD1 G93A
		[62]	Patient prefrontal cortex	NA
Glyceraldehyde-3-Phosphate Dehydrogenase (GAPDH)	3	[54]	Mouse spinal cord	SOD1 G93A
		[53]	Mouse muscle	Wobbler mouse
		[49]	Mouse spinal cord	Wobbler mouse
Valosin Containing Protein (VCP)	2	[54]	Mouse spinal cord	SOD1 G93A
		[46]	Patient skeletal muscle	NA
Septin 9 (SEPT9)	2	[50]	Mouse ventral roots	SOD1 G93A
		[44]	SH-SY5Y cells	TDP-43 Loss
Malate Dehydrogenase 1 (MDH1)	2	[54]	Mouse spinal cord	SOD1 G93A
		[52]	Mouse spinal cord	SOD1 G127X
Actin, Alpha Cardiac Muscle 1 (ACTC1)	2	[53]	Mouse muscle	Wobbler mouse
		[44]	SH-SY5Y cells	TDP-43 Loss
NADH dehydrogenase [ubiquinone] iron-sulfur protein 8, mitochondrial (NDUFS8)	2	[57]	NSC34 cells (mitochondrial fraction)	SOD1 G93A
		[61]	Patient spinal cord	NA
Calumenin (CALU)	2	[50]	Mouse ventral roots	SOD1 G93A
		[44]	SH-SY5Y cells	TDP-43 Loss
Creatine Kinase, Mitochondrial 1 (CKMT1B)	2	[54]	Mouse spinal cord	SOD1 G93A
		[49]	Mouse spinal cord	Wobbler mouse
Glutamate Dehydrogenase (GLUD1)	2	[54]	Mouse spinal cord	SOD1 G93A
		[49]	Mouse spinal cord	Wobbler mouse
Apolipoprotein E (APOE)	2	[56]	Mouse spinal cord	SOD1 G93A
		[50]	Mouse ventral roots	SOD1 G93A
Enoyl Coenzyme A Hydratase, Short Chain 1 (ECHS1)	2	[55]	Mouse spinal cord (pre-symptomatic)	SOD1 G93A
		[47]	Mouse astrocytes	SOD1 G93A
Single Stranded DNA Binding Protein 1 (SSBP1)	2	[58]	Rat spinal cord (mitochondrial fraction)	SOD1 G93A
		[44]	SH-SY5Y cells	TDP-43 Loss
Glycerol Phosphate Dehydrogenase 2 (GPD2)	2	[54]	Mouse spinal cord	SOD1 G93A
		[49]	Mouse spinal cord	Wobbler mouse

Aldolase C (ALDOC)	2	[54]	Mouse spinal cord	SOD1 G93A
		[47]	Mouse astrocytes	SOD1 G93A
Glutamine Ammonia Ligase (GLUL)	2	[54]	Mouse spinal cord	SOD1 G93A
		[49]	Mouse spinal cord	Wobbler mouse
Endoplasmic Reticulum Protein 29 (ERP29)	2	[52]	Mouse spinal cord	SOD1 G127X
		[48]	Patient blood mononuclear cells	NA
Isocitrate Dehydrogenase 2 (IDH2)	2	[54]	Mouse spinal cord	SOD1 G93A
		[58]	Rat spinal cord (mitochondrial fraction)	SOD1 G93A
Lactate Dehydrogenase B (LDHB)	2	[54]	Mouse spinal cord	SOD1 G93A
		[48]	Patient blood mononuclear cells	NA
Decreased expression				
Neurosecretory protein VGF (VGF)	2	[44]	SH-SY5Y cells	TDP-43 Loss
		[62]	Patient prefrontal cortex	NA
Myosin Light Chain, Phosphorylatable, Fast Skeletal Muscle (MYLPF)	2	[50]	Mouse ventral roots	SOD1 G93A
		[45]	Patient skeletal muscle	NA
Nudix Motif 2 (NUDT2)	2	[52]	Mouse spinal cord	SOD1 G127X
		[43]	Rat spinal cord (mitochondrial fraction)	Patient CSF injected
Adenylate Kinase 2 (AK2)	2	[57]	NSC34 cells (mitochondrial fraction)	SOD1 G93A
		[58]	Rat spinal cord (mitochondrial fraction)	SOD1 G93A
Far Upstream Element-Binding Protein 1 (FUBP1)	2	[48]	Patient blood mononuclear cells	NA
		[44]	SH-SY5Y cells	TDP-43 Loss
Filamin A (FLNA)	2	[48]	Patient blood mononuclear cells	NA
		[47]	Mouse astrocytes	SOD1 G93A
Golgin B1 (GOLGB1)	2	[48]	Patient blood mononuclear cells	NA
		[44]	SH-SY5Y cells	TDP-43 Loss
Heat Shock Protein Family D, Member 1 (HSPD1)	2	[58]	Rat spinal cord (mitochondrial fraction)	SOD1 G93A
		[44]	SH-SY5Y cells	TDP-43 Loss
Acyl- Coenzyme A Dehydrogenase, Medium Chain (ACADM)	2	[57]	NSC34 cells (mitochondrial fraction)	SOD1 G93A
		[46]	Patient skeletal muscle	NA
Nadh Dehydrogenase 1 Beta Subcomplex Subunit 10 (NDUFB10)	2	[57]	NSC34 cells (mitochondrial fraction)	SOD1 G93A
		[44]	SH-SY5Y cells	TDP-43 Loss
Solute Carrier Family 25, Member 4 (SLC25A4)	2	[58]	Rat spinal cord (mitochondrial fraction)	SOD1 G93A

		[44]	SH-SY5Y cells	TDP-43 Loss
Ubiquinol-Cytochrome C Reductase Core Protein I (UQCRC1)	2	[47]	Mouse astrocytes	SOD1 G93A
		[44]	SH-SY5Y cells	TDP-43 Loss
Chaperonin Containing Tcp1 Subunit 6a (CCT6A)	2	[57]	NSC34 cells (mitochondrial fraction)	SOD1 G93A
		[44]	SH-SY5Y cells	TDP-43 Loss
2',3'-Cyclic Nucleotide 3 Phosphodiesterase (CNP)	2	[52]	Mouse spinal cord	SOD1 G127X
		[44]	SH-SY5Y cells	TDP-43 Loss

Proteins that showed consistent direction of differential expression in cells and tissues in at least two proteomic studies of ALS. Proteins are listed according to direction of differential expression (proteins with increased expression are listed first, followed by proteins with decreased expression). Protein name is given, followed by official gene symbol in brackets. ALS model and sample type used in each study are listed, together with the number of studies that identified protein changes (studies are listed in the reference column).

Table S5. Proteins that showed contradictory direction of differential expression in cells and tissues across two or more proteomic studies of ALS.

Protein (Official Gene Symbol)	Increased Expression	Decreased Expression
Prolyl 4-Hydroxylase Subunit Beta (P4HB)	[48,50,54,55]	[47]
Phosphoglycerate Mutase 1 (PGAM1)	[48,49,52]	[44,55,60]
Peptidylprolyl Isomerase A (PPIA)	[48,54,55]	[47]
Dihydropyrimidinase-Like 2 (DPYSL2)	[49,54]	[47,52,60]
Pyruvate Kinase, Muscle (PKM)	[49,54]	[47,60]
NADH Dehydrogenase Fe-S Protein 1 (NDUFS1)	[54]	[58,60]
Enolase 1 (ENO1)	[54,55]	[47,48]
ATP Synthase, H+ Transporting, Mitochondrial F1 Complex, Alpha Subunit 1 (ATP5A1)	[54]	[47,57,60]
Tubulin Alpha 1b (TUBA1B)	[44,60]	[57]
Voltage-Dependent Anion Channel 1 (VDAC1)	[47]	[52,57]
Transketolase (TKT)	[44,49]	[47]
Pyruvate Dehydrogenase E1 Alpha 1 (PDHA1)	[54,55]	[57]
4-Aminobutyrate Aminotransferase (ABAT)	[49,58]	[52]
Atp Synthase, H+ Transporting, Mitochondrial F1 Complex, Beta Polypeptide (ATP5B)	[48,60]	[44,48]
Aconitase 2 (ACO2)	[49,54,57]	[57]
Vimentin (VIM)	[44,47,54]	[47]
Heat Shock Protein Family A, Member 5 (HSPA5)	[61]	[47,48,57]
Actin Beta (ACTB)	[47,48]	[62]
Phosphoglycerate kinase 1 (PGK1)	[48,49,60]	[48,49]
Tubulin beta-2B chain (TUBB2B)	[44,60]	[62]
Glutathione S-transferase Mu 1 (GSTM1)	[62]	[59]
Dipeptidyl peptidase 3 (DPP3)	[61]	[62]
Prohibitin (PHB)	[47]	[58]
Tubulin, Beta 4b (TUBB4B)	[47]	[44]
Electron Transferring Flavoprotein, Alpha (ETFA)	[55]	[52]
Actin, Gamma, Cytoplasmic 1 (ACTG1)	[52]	[57]
Annexin A5 (ANXA5)	[54]	[52]
Creatine Kinase, Muscle (CKM)	[53]	[50,53]
Crystallin, Alpha B (CRYAB)	[54]	[47]
Growth Factor Receptor-Bound Protein 2 (GRB2)	[44]	[52]
Hexosaminidase Subunit Alpha (HEXA)	[50]	[43]
Heat Shock Protein 90 Alpha Family Class A Member 1 (HSP90AA1)	[54]	[44]
Isocitrate Dehydrogenase 3 (NAD+) Beta (IDH3B)	[58]	[57]
Heat Shock Protein 90, Beta (Grp94), Member 1 (HSP90B1)	[54]	[47]
Mitogen-Activated Protein Kinase 1 (MAPK1)	[54]	[47]
Lon Peptidase 1 (LONP1)	[44]	[57]
Dynamin 1 (DNM1)	[52,62]	[60]
2,4-Dienoyl Coa Reductase 1 (DECR1)	[58]	[44]

Proteins that showed contradictory direction of differential expression in cells and tissues in at least two proteomic studies of ALS. Protein name is given, followed by official gene symbol in brackets. Studies that identified increased or decreased protein expression are listed in column two and three.

Table S6. GO term analysis of proteins that were differentially expressed in tissues and cells in ALS proteomic studies.

Enriched Term	Count	Proteins
		Biological process
Programmed cell death	15	ACTC1, APOE, CALR, ERP29, FLNA, GAPDH, HSPB1, HSPD1, PRDX2, SOD1, SOD2, YWHAG, VCP, SLC25A4, NUDT2
Protein transport and localization	13	VGF, APOE, CALR, CCT6A, ERP29, FLNA, GLUD1, GLUL, HSPA8, HSPD1, SLC25A4, YWHAG, VCP
Nucleotide metabolism	13	CNP, NDUFS8, NDUFB10, AK2, ALDOA, ALDOC, APOE, GAPDH, HSPA8, LDHB, MDH1, UQCRC1, VCP
Carbohydrate derivative metabolism	11	NDUFS8, NDUFB10, AK2, ALDOA, ALDOC, APOE, GAPDH, GDP2, HSPA8, UQCRC1, VCP
Organic acid metabolism	10	ACADM, ALDOA, ALDOC, ECHS1, GLUD1, GLUL, GAPDH, IDH2, LDHB, MDH1
Protein complex assembly	10	NDUFS8, NDUFB10, ALDOA, CALR, GLUL, HSPA8, HSPD1, SEPT9, SOD2, VCP
Cytoskeleton organization	9	CNP, ACTC1, ALDOA, APOE, CALR, FLNA, GFAP, GAPDH, SOD1
Response to oxidative stress	9	NDUFS8, APOE, HSPB1, HSPD1, NUDT2, PRDX2, PRDX6, SOD1, SOD2
ATP metabolism	9	NDUFS8, NDUFB10, AK2, ALDOA, ALDOC, GAPDH, HSPA8, UQCRC1, VCP
Cell adhesion	9	ALDOA, CALR, FLNA, HSPA8, HSPB1, HSPD1, PRDX6, SEPT9, SOD1
Carbohydrate metabolism	9	ACADM, ALDOA, ALDOC, GAPDH, GPD2, IDH2, LDHB, MDH1, VCP
Mitochondrion organization	8	CNP, NDUFS8, NDUFB10, HSPD1, SSBP1, SLC25A4, SOD2, YWHAG
Cellular amide metabolic process	7	APOE, CALR, GAPDH, HSPB1, SLC25A4, SOD1, SOD2
Cellular respiration	7	NDUFS8, NDUFB10, IDH2, MDH1, SOD2, UQCRC1, VCP
Synaptic signaling	7	CNP, VGF, APOE, GFAP, GLUL, HSPA8, YWHAG
Protein stability	6	CALR, CCT6A, FLNA, GAPDH, HSPA8, HSPD1
Protein secretion	6	VGF, ERP29, GLUD1, GLUL, HSPD1, SLC25A4
NADH metabolism	6	ALDOA, ALDOC, GAPDH, LDHB, MDH1, VCP
Response to wounding	6	APOE, FLNA, GFAP, HSPB1, SOD1, SOD2
Response to unfolded protein	5	CALR, HSPA8, HSPB1, HSPD1, VCP
Gluconeogenesis	5	ALDOA, ALDOC, GAPDH, GPD2, MDH1
Regulation of protein kinase activity	5	APOE, ERP29, HSPB1, SOD1, YWHAG
Muscle contraction	5	ACTC1, ALDOA, FLNA, MYLPF, SOD1
Response to drug	5	ACTC1, CALR, HSPD1, SOD1, SOD2
Blood circulation	5	ACTC1, APOE, FLNA, SOD1, SOD2
Response to temperature stimulus	5	VGF, ACADM, HSPD1, SOD1, SOD2
Striated muscle tissue development	4	ACTC1, ACADM, CALR, MYLPF
Hormone secretion	4	VGF, GLUD1, GLUL, SLC25A4
Lipid catabolic process	4	ACADM, APOE, ECHS1, PRDX6
Aging	4	CNP, CALR, SOD1, SOD2
Glycolytic process	3	ALDOA, ALDOC, GAPDH
Cardiac muscle cell differentiation	3	ACTC1, ACADM, CALR

Response to starvation	3	ACADM, GLUL, HSPA8
		Cellular component
Extracellular vesicle	28	CNP, NDUFB10, ACTC1, ACADM, AK2, ALDOA, ALDOC, APOE, CALR, CCT6A, ERP29, ECHS1, FLNA, GLUL, GAPDH, HSPA8, HSPB1, HSPD1, IDH2, LDHB, MDH1, PRDX2, PRDX6, SSBP1, SOD1, SOD2, YWHAG, VCP
Mitochondrion	21	CNP, NDUFS8, NDUFB10, ACADM, AK2, ALDOC, ECHS1, GLUD1, GLUL, GDP2, HSPD1, IDH2, LDHB, MDH1, NUDT2, SSBP1, SLC25A4, SOD1, SOD2, YWHAG, UQCRC1
Cytosol	21	ACTC1, ALDOA, ALDOC, CALR, CCT6A, FLNA, GFAP, GLUL, GAPDH, HSPA8, HSPB1, HSPD1, IDH2, LDHB, MDH1, MYLPF, PRDX2, PRDX6, SOD1, YWHAG, VCP
Myelin sheath	13	CNP, GFAP, GLUL, HSPA8, HSPD1, LDHB, MDH1, SLC25A4, SOD1, SOD2, YWHAG, UQCRC1, VCP
Cytoplasmic vesicle	12	CNP, VGF, ALDOA, APOE, CALR, CALU, ERP29, HSPA8, HSPD1, PRDX6, SOD1, YWHAG
Cytoskeleton	12	CNP, ACTC1, ALDOA, ALDOC, CCT6A, FLNA, GFAP, GAPDH, HSPB1, MDH1, MYLPF, SEPT9
Extracellular space	12	CNP, VGF, ACTC1, ALDOA, APOE, CALR, HSPA8, HSPB1, HSPD1, MDH1, PRDX6, SOD1
Cell projection	10	CNP, ACTC1, ACADM, AK2, APOE, FLNA, GFAP, GLUL, SEPT9, SOD1
Cell junction	9	ACTC1, ALDOA, CALR, FLNA, HSPA8, HSPB1, PRDX6, SEPT9, YWHAG
Extracellular matrix	9	APOE, CALR, CCT6A, FLNA, GAPDH, HSPA8, HSPB1, HSPD1, SOD1
Perinuclear region of cytoplasm	6	CNP, CALR, FLNA, GAPDH, SEPT9, VCP
Cell body	6	ACTC1, APOE, FLNA, GFAP, GLUL, SOD1
Contractile fiber	5	ACTC1, ALDOA, FLNA, HSPB1, MYLPF
Lysosome	5	GFAP, HSPA8, MYLPF, PRDX6, SOD1
Peroxisome	4	ACADM, HSPD1, IDH2, SOD1
Oxidoreductase complex	4	NDUFS8, NDUFB10, GDP2, UQCRC1
Pigment granule	4	CNP, CALU, ERP29, HSPA8
Respiratory chain complex	3	NDUFS8, NDUFB10, UQCRC1
		Molecular function
Catalytic activity	27	CNP, NDUFS8, NDUFB10, ACTC1, ACADM, AK2, ALODA, ALDOC, ERP29, ECHS1, GLUD1, GLUL, GAPDH, GPD2, HSPA8, HSPD1, IDH2, LDHB, MDH1, NUDT2, PRDX2, PRDX6, SEPT9, SOD1, SOD2, UQCRC1, VCP
Nucleotide binding	16	CNP, ACTC1, ACADM, AK2, CCT6A, GLUD1, GLUL, GAPDH, HSPA8, HSPD1, IDH2, LDHB, MDH1, NUDT2, SEPT9, VCP
Carbohydrate derivative binding	14	ACTC1, AK2, APOE, CALR, CCT6A, FLNA, GFAP, GLUD1, GLUL, HSPA8, HSPD1, NUDT2, SEPT9, VCP
Identical protein binding	14	ACADM, ALDOA, APOE, ERP29, FLNA, GFAP, GLUD1, GLUL, GAPDH, HSPB1, LDHB, SOD1, SOD2, VCP
Oxidoreductase activity	14	NDUFS8, NDUFB10, ACADM, GLUD1, GAPDH, GDP2, IDH2, LDHB, MDH1, PRDX2, PRDX6, SOD1, SOD2, UQCRC1
RNA and DNA binding	13	CNP, ALDOA, CALR, CCT6A, FUBP1, FLNA, GOLGB1, HSPA8, HSPB1, HSPD1, SSBP1, YWHAG, VCP
Enzyme binding	12	CALR, FLNA, GFAP, HSPA8, HSPB1, HSPD1, LDHB, PRDX6, SOD1, YWHAG, UQCRC1, VCP
Cell adhesion molecule binding	7	ALDOA, CALR, FLNA, GFAP, HSPA8, PRDX6, SEPT9
Cytoskeletal protein binding	6	ACTC1, ALDOA, ALDOC, APOE, FLNA, GAPDH

Coenzyme binding	6	ACADM, GLUD1, GAPDH, IDH2, LDHB, MDH1
Antioxidant activity	5	APOE, PRDX2, PRDX6, SOD1, SOD2
Unfolded protein binding	4	CALR, CCT6A, HSPA8, HSPD1
Chaperone binding	4	CALR, ERP29, HSPD1, SOD1
Glycoprotein binding	3	CALR, FLNA, GFAP

Enriched terms connected to “Biological process”, “Cellular component” and “Molecular function” are presented together with the number of annotated proteins. Proteins are listed in column three.

Table S7. GO term analysis of proteins that were differentially expressed in SMA and ALS proteomic studies.

Enriched term	Count	Proteins
	Biological process	
Programmed cell death	10	ANXA5, CALR, HSP90B1, HSPD1, PRDX2, SOD1, VDAC1, GAPDH, HSP90AA1, YWHAG
Establishment of localization	10	CNP, ATP5A1, ALDOA, CALR, HSP90AA1, HSP90B1, HSPD1, SOD1, YWHAG, VDAC1
Response to chemical	10	ANXA5, CALR, HSP90B1, HSPD1, PRDX2, SOD1, CNP, GAPDH, HSP90AA1, YWHAG
Regulation of protein metabolism	7	CALR, HSP90B1, HSP90AA1, YWHAG, GAPDH, HSPD1, SOD1
Cytoskeleton organization	7	ALDOA, CALR, HSP90B1, SOD1, VIM, CNP, GAPDH
Nervous system development	6	CNP, CALR, SOD1, HSP90AA1, YWHAG, VIM
Regulation of immune system	6	CALR, HSP90B1, HSPD1, SOD1, GAPDH, HSP90AA1
Vesicle-mediated transport	5	ALDOA, CALR, HSP90B1, SOD1, HSP90AA1
Metabolism of reactive oxygen species	5	HSPD1, PRDX2, SOD1, VDAC1, HSP90AA1
Oxidation-reduction process	5	ALDOA, PRDX2, PGK1, SOD1, GAPDH
Mitochondrion organization	5	CNP, HSP90AA1, HSPD1, YWHAG, VDAC1
Mitochondrial transport	5	CNP, ATP5A1, HSP90AA1, YWHAG, VDAC1
Cellular homeostasis	5	ALDOA, CALR, HSP90B1, PRDX2, SOD1
Nucleotide metabolism	5	ALDOA, GAPDH, PGK1, ATP5A1, CNP
Intracellular protein transport	4	CALR, HSP90B1, HSP90AA1, YWHAG
Epithelial cell differentiation	4	PGK1, SOD1, VIM, VDAC1
Response to unfolded protein	4	CALR, HSP90B1, HSPD1, HSP90AA1
ATP metabolism	4	ALDOA, GAPDH, PGK1, ATP5A1
Muscle contraction	4	ALDOA, SOD1, VIM, HSP90AA1
Glucose metabolism	3	ALDOA, GAPDH, PGK1
Response to ATP	3	HSP90B1, HSPD1, SOD1
NADH metabolism	3	ALDOA, GAPDH, PGK1
Response to temperature stimulus	3	HSPD1, HSP90AA1, SOD1
Aging	3	CALR, SOD1, CNP
Autophagy	3	VDAC1, GAPDH, HSP90AA1
	Cellular component	
Extracellular vesicle	15	ALDOA, ANXA5, CALR, HSP90B1, HSPD1, PRDX2, PGK1, SOD1, VIM, VDAC1, GAPDH, HSP90AA1, YWHAG, ATP5A1, CNP
Plasma membrane	11	CNP, ATP5A1, ANXA5, CALR, GAPDH, HSP90AA1, HSP90B1, HSPD1, SOD1, VIM, VDAC1
Cytosol	11	ALDOA, CALR, HSP90B1, HSPD1, PRDX2, PGK1, SOD1, VIM, GAPDH, HSP90AA1, YWHAG
Nucleus	10	CNP, ATP5A1, ALDOA, CALR, GAPDH, HSP90AA1, HSP90B1, HSPD1, SOD1, VDAC1
Cytoplasmic vesicle	9	ALDOA, CALR, HSP90B1, HSPD1, SOD1, VDAC1, CNP, HSP90AA1, YWHAG
Extracellular matrix	8	CALR, HSP90B1, HSPD1, SOD1, VIM, ATP5A1, GAPDH, HSP90AA1
Protein complex	8	ATP5A1, CALR, GAPDH, HSP90AA1, HSP90B1, HSPD1, SOD1, VDAC1
Myelin sheath	7	HSPD1, VDAC1, HSP90AA1, YWHAG, ATP5A1, CNP, SOD1
Extracellular space	6	ALDOA, ANXA5, CALR, HSPD1, SOD1, CNP

Mitochondrion	6	CNP, ATP5A1, HSPD1, SOD1, YWHAG, VDAC1
Cell junction	6	ALDOA, ANXA5, CALR, HSP90B1, VIM, YWHAG
Perinuclear region of cytoplasm	5	CNP, CALR, GAPDH, HSP90AA1, HSP90B1
Peroxisome	3	HSPD1, SOD1, VIM
Membrane microdomain	3	HSPD1, PGK1, VDAC1
Melanosome	3	CNP, HSP90AA1, HSP90B1
	Molecular function	
RNA binding	9	CNP, ATP5A1, ALDOA, CALR, HSP90AA1, HSP90B1, HSPD1, YWHAG, VIM
Enzyme binding	7	CALR, HSP90AA1, HSP90B1, HSPD1, SOD1, YWHAG, VDAC1
Carbohydrate derivative binding	7	ATP5A1, CALR, HSP90AA1, HSP90B1, HSPD1, PGK1, VIM
Nucleotide binding	7	CNP, ATP5A1, GAPDH, HSP90AA1, HSP90B1, HSPD1, PGK1
ATP binding	5	ATP5A1, HSP90AA1, HSP90B1, HSPD1, PGK1
Receptor binding	5	ATP5A1, CALR, HSP90AA1, HSP90B1, YWHAG
Unfolded protein binding	4	CALR, HSP90AA1, HSP90B1, HSPD1
Protein complex binding	4	CALR, HSP90AA1, VIM, VDAC1
Phosphatase binding	3	HSP90AA1, HSP90B1, SOD1
Glycoprotein binding	3	CALR, HSP90AA1, VIM
Chaperone binding	3	CALR, HSPD1, SOD1
ATPase activity	3	ATP5A1, HSP90AA1, HSPD1

Enriched terms connected to "Biological process", "Cellular component" and "Molecular function" are presented together with the number of annotated proteins. Proteins are listed in column three.

References (these align with the reference list in the main manuscript but are given here again for ease of reference):

20 Chen, Y.; Liu, X.H.; Wu, J.-J.; Ren, H.M.; Wang, J.; Ding, Z.T.; Jiang, V.P. Proteomic analysis of cerebrospinal fluid in amyotrophic lateral sclerosis. *Exp. Ther. Med.* **2016**, *11*, 2095–2106, doi:10.3892/etm.2016.3210.

31. Collins, M.A.; An, J.; Hood, B.L.; Conrads, T.P.; Bowser, R.P. Label-Free LC–MS/MS Proteomic Analysis of Cerebrospinal Fluid Identifies Protein/Pathway Alterations and Candidate Biomarkers for Amyotrophic Lateral Sclerosis. *J. Proteome Res.* **2015**, *14*, 4486–4501, doi:10.1021/acs.jproteome.5b00804.
32. Varghese, A.M.; Sharma, A.; Mishra, P.; Vijayalakshmi, K.; Harsha, H.C.; Sathyaprabha, T.N.; Bharath, S.M.; Nalini, A.; Alladi, P.A.; Raju, T.R. Chitotriosidase-a putative biomarker for sporadic amyotrophic lateral sclerosis. *Clin. Proteomics* **2013**, *10*, 19, doi:10.1186/1559-0275-10-19.
33. Mendonça, D.M.F.; Pizzati, L.; Mostacada, K.; de Martins, S.C.; Higashi, R.; Ayres Sá, L.; Moura Neto, V.; Chimelli, L.; Martinez, A.M.B. Neuroproteomics: An insight into ALS. *Neurol. Res.* **2012**, *34*, 937–943, doi:10.1179/1743132812Y.0000000092.
34. Von Neuhoff, N.; Oumeraci, T.; Wolf, T.; Kollewe, K.; Bewerunge, P.; Neumann, B.; Brors, B.; Bufler, J.; Wurster, U.; Schlegelberger, B.; et al. Monitoring CSF Proteome Alterations in Amyotrophic Lateral Sclerosis: Obstacles and Perspectives in Translating a Novel Marker Panel to the Clinic. *PLoS ONE* **2012**, *7*, e44401, doi:10.1371/journal.pone.0044401.
35. Ryberg, H.; An, J.; Darko, S.; Lustgarten, J.L.; Jaffa, M.; Gopalakrishnan, V.; Lacomis, D.; Cudkowicz, M.; Bowser, R. Discovery and verification of amyotrophic lateral sclerosis biomarkers by proteomics. *Muscle Nerve* **2010**, *42*, 104–111, doi:10.1002/mus.21683.
36. Brettschneider, J.; Mogel, H.; Lehmensiek, V.; Ahlert, T.; Süssmuth, S.; Ludolph, A.C.; Tumani, H. Proteome analysis of cerebrospinal fluid in amyotrophic lateral sclerosis (ALS). *Neurochem. Res.* **2008**, *33*, 2358–2363, doi:10.1007/s11064-008-9742-5.
37. Ranganathan, S.; Williams, E.; Ganchev, P.; Gopalakrishnan, V.; Lacomis, D.; Urbinelli, L.; Newhall, K.; Cudkowicz, M.E.; Brown, R.H., Jr.; Bowser, R. Proteomic profiling of cerebrospinal fluid identifies biomarkers for amyotrophic lateral sclerosis. *J. Neurochem.* **2005**, *95*, 1461–1471, doi:10.1111/j.1471-4159.2005.03478.x.
38. Ranganathan, S.; Nicholl, G.C.B.; Henry, S.; Lutka, F.; Sathanoori, R.; Lacomis, D.; Bowser, R. Comparative proteomic profiling of cerebrospinal fluid between living and post mortem ALS and control subjects. Amyotroph. *Lateral Scler.* **2007**, *8*, 373–379, doi:10.1080/17482960701549681.
39. Pasinetti, G.M.; Ungar, L.H.; Lange, D.J.; Yemul, S.; Deng, H.; Yuan, X.; Brown, R.H.; Cudkowicz, M.E.; Newhall, K.; Peskind, E.; et al. Identification of potential CSF biomarkers in ALS. *Neurology* **2006**, *66*, 1218–1222, doi:10.1212/01.wnl.0000203129.82104.07.
40. Thompson, A.G.; Gray, E.; Thezenas, M.L.; Charles, D.; Evetts, S.; Hu, M.T.; Talbot, K.; Fischer, R.; Kessler, B.M.; Turner, M.R. Cerebrospinal Fluid Macrophage Biomarkers in Amyotrophic Lateral Sclerosis. *Ann. Neurol.* **2018**, *83*, 258–268, doi:10.1002/ana.25143.
41. De Benedetti, S.; Gianazza, E.; Banfi, C.; Marocchi, A.; Lunetta, C.; Penco, S.; Bonomi, F.; Iametti, S. Serum Proteome in a Sporadic Amyotrophic Lateral Sclerosis Geographical Cluster. *Proteomics Clin. Appl.* **2017**, *11*, 1700043, doi:10.1002/prca.201700043.
42. Schwenk, B.M.; Hartmann, H.; Serdaroglu, A.; Schludi, M.H.; Hornburg, D.; Meissner, F.; Orozco, D.; Colombo, A.; Tahirovic, S.; Michaelsen, M.; et al. TDP-43 loss of function inhibits endosomal trafficking and alters trophic signaling in neurons. *EMBO J.* **2016**, *35*, 2350–2370, doi:10.15252/embj.201694221.
43. Sharma, A.; Varghese, A.M.; Vijaylakshmi, K.; Sumitha, R.; Prasanna, V.K.; Shruthi, S.; Chandrasekhar Sagar, B.K.; Datta, K.K.; Gowda, H.; Nalini, A.; et al. Cerebrospinal Fluid from Sporadic Amyotrophic Lateral Sclerosis Patients Induces Mitochondrial and Lysosomal Dysfunction. *Neurochem. Res.* **2016**, *41*, 965–984, doi:10.1007/s11064-015-1779-7.
44. Stalekar, M.; Yin, X.; Rebolj, K.; Darovic, S.; Troakes, C.; Mayr, M.; Shaw, C.E.; Rogelj, B. Proteomic analyses reveal that loss of TDP-43 affects RNA processing and intracellular transport. *Neuroscience* **2015**, *293*, 157–170, doi:10.1016/j.neuroscience.2015.02.046.
45. Conti, A.; Riva, N.; Pesca, M.; Iannaccone, S.; Cannistraci, C.V.; Corbo, M.; Previtali, S.C.; Quattrini, A.; Alessio, M. Increased expression of Myosin binding protein H in the skeletal muscle of amyotrophic lateral sclerosis patients. *Biochim. Biophys. Acta* **2014**, *1842*, 99–106, doi:10.1016/j.bbadis.2013.10.013.
46. Elf, K.; Shevchenko, G.; Nygren, I.; Larsson, L.; Bergquist, J.; Askmark, H.; Artemenko, K. Alterations in

muscle proteome of patients diagnosed with amyotrophic lateral sclerosis. *J. Proteomics* **2014**, *108*, 55–64, doi:10.1016/j.jprot.2014.05.004.

47. Basso, M.; Pozzi, S.; Tortarolo, M.; Fiordaliso, F.; Bisighini, C.; Pasetto, L.; Spaltro, G.; Lidonnici, D.; Gensano, F.; Battaglia, E.; et al. Mutant Copper-Zinc superoxide dismutase (SOD1) induces protein secretion pathway alterations and exosome release in astrocytes: Implications for disease spreading and motor neuron pathology in amyotrophic lateral sclerosis. *J. Biol. Chem.* **2013**, *288*, 15699–15711, doi:10.1074/jbc.M112.425066.
48. Nardo, G.; Pozzi, S.; Pignataro, M.; Lauranzano, E.; Spano, G.; Garbelli, S.; Mantovani, S.; Marinou, K.; Papetti, L.; Monteforte, M.; et al. Amyotrophic lateral sclerosis multiprotein biomarkers in peripheral blood mononuclear cells. *PLoS ONE* **2011**, *6*, e25545, doi:10.1371/journal.pone.0025545.
49. Bastone, A.; Fumagalli, E.; Bigini, P.; Perini, P.; Bernardinello, D.; Cagnotto, A.; Mereghetti, I.; Curti, D.; Salmona, M.; Mennini, T. Proteomic profiling of cervical and lumbar spinal cord reveals potential protective mechanisms in the wobbler mouse, a model of motor neuron degeneration. *J. Proteome Res.* **2009**, *8*, 5229–5240, doi:10.1021/pr900569d.
50. Zhou, J.-Y.; Afjehi-Sadat, L.; Asress, S.; Duong, D.M.; Cudkowicz, M.; Glass, J.D.; Peng, J. Galectin-3 is a candidate biomarker for ALS: Discovery by a proteomics approach. *J. Proteome Res.* **2010**, *9*, 5133–5141, doi:10.1021/pr100409r.
51. Duplan, L.; Bernard, N.; Casseron, W.; Dudley, K.; Thouvenot, E.; Honnorat, J.; Rogemond, V.; De Bovis, B.; Aebischer, P.; Marin, P.; et al. Collapsin response mediator protein 4a (CRMP4a) is upregulated in motoneurons of mutant SOD1 mice and can trigger motoneuron axonal degeneration and cell death. *J. Neurosci.* **2010**, *30*, 785–796, doi:10.1523/JNEUROSCI.5411-09.2010.
52. Bergemalm, D.; Forsberg, K.; Jonsson, P.A.; Graffmo, K.S.; Brännströ, T.; Andersen, P.M.; Antti, H.; Marklund, S.L. Changes in the spinal cord proteome of an amyotrophic lateral sclerosis murine model determined by differential in-gel electrophoresis. *Mol. Cell. Proteomics* **2009**, *8*, 1306–1317, doi:10.1074/mcp.M900046-MCP200.
53. Staunton, L.; Jockusch, H.; Ohlendieck, K. Proteomic analysis of muscle affected by motor neuron degeneration: The wobbler mouse model of amyotrophic lateral sclerosis. *Biochem. Biophys. Res. Commun.* **2011**, *406*, 595–600, doi:10.1016/j.bbrc.2011.02.099.
54. Basso, M.; Samengo, G.; Nardo, G.; Massignan, T.; D'Alessandro, G.; Tartari, S.; Cantoni, L.; Marino, M.; Cheroni, C.; De Biasi, S.; et al. Characterization of detergent-insoluble proteins in ALS indicates a causal link between nitrative stress and aggregation in pathogenesis. *PLoS ONE* **2009**, *4*, e8130, doi:10.1371/journal.pone.0008130.
55. Massignan, T.; Casoni, F.; Basso, M.; Stefanazzi, P.; Biasini, E.; Tortarolo, M.; Salmona, M.; Gianazza, E.; Bendotti, C.; Bonetto, V. Proteomic analysis of spinal cord of presymptomatic amyotrophic lateral sclerosis G93A SOD1 mouse. *Biochem. Biophys. Res. Commun.* **2007**, *353*, 719–725, doi:10.1016/j.bbrc.2006.12.075.
56. Strey, C.W.; Spellman, D.; Stieber, A.; Gonatas, J.O.; Wang, X.; Lambris, J.D.; Gonatas, N.K. Dysregulation of stathmin, a microtubule-destabilizing protein, and up-regulation of Hsp25, Hsp27, and the antioxidant peroxiredoxin 6 in a mouse model of familial amyotrophic lateral sclerosis. *Am. J. Pathol.* **2004**, *165*, 1701–1718, doi:10.1016/S0002-9440(10)63426-8.
57. Fukada, K.; Zhang, F.; Vien, A.; Cashman, N.R.; Zhu, H. Mitochondrial Proteomic Analysis of a Cell Line Model of Familial Amyotrophic Lateral Sclerosis. *Mol. Cell. Proteomics* **2004**, *3*, 1211–1223, doi:10.1074/mcp.M400094-MCP200.
58. Li, Q.; Vande Velde, C.; Israelson, A.; Xie, J.; Bailey, A.O.; Dong, M.-Q.; Chun, S.-J.; Roy, T.; Winer, L.; Yates, J.R.; et al. ALS-linked mutant superoxide dismutase 1 (SOD1) alters mitochondrial protein composition and decreases protein import. *Proc. Natl. Acad. Sci. USA* **2010**, *107*, 21146–21151, doi:10.1073/pnas.1014862107.
59. Allen, S.; Heath, P.R.; Kirby, J.; Wharton, S.B.; Cookson, M.R.; Menzies, F.M.; Banks, R.E.; Shaw, P.J. Analysis of the cytosolic proteome in a cell culture model of familial amyotrophic lateral sclerosis reveals alterations to the proteasome, antioxidant defenses, and nitric oxide synthetic pathways. *J. Biol. Chem.* **2003**, *278*, 6371–6383, doi:10.1074/jbc.M209915200.
60. Shin, J.-H.; London, J.; Le Pecheur, M.; Weitzdoerfer, R.; Hoeger, H.; Lubec, G. Proteome analysis in hippocampus of mice overexpressing human Cu/Zn-superoxide dismutase 1. *Neurochem. Int.* **2005**, *46*, 641–653, doi:10.1016/j.neuint.2004.06.017.

61. Engelen-Lee, J.; Blokhuis, A.M.; Spliet, W.G.M.; Pasterkamp, R.J.; Aronica, E.; Demmers, J.A.A.; Broekhuizen, R.; Nardo, G.; Bovenschen, N.; Van Den Berg, L.H. Proteomic profiling of the spinal cord in ALS: Decreased ATP5D levels suggest synaptic dysfunction in ALS pathogenesis. *Amyotroph. Lateral Scler. Front. Degener.* **2017**, *18*, 210–220, doi:10.1080/21678421.2016.1245757.
62. Umoh, M.E.; Dammer, E.B.; Dai, J.; Duong, D.M.; Lah, J.J.; Levey, A.I.; Gearing, M.; Glass, J.D.; Seyfried, N.T. A proteomic network approach across the ALS-FTD disease spectrum resolves clinical phenotypes and genetic vulnerability in human brain. *EMBO Mol. Med.* **2018**, *10*, 48–62, doi:10.15252/emmm.201708202.
